# Functional interplay between TFIIH and KAT2A regulates higher-order chromatin structure and class II gene expression

**DOI:** 10.1038/s41467-019-09270-2

**Published:** 2019-03-20

**Authors:** Jérémy Sandoz, Zita Nagy, Philippe Catez, Gizem Caliskan, Sylvain Geny, Jean-Baptiste Renaud, Jean-Paul Concordet, Arnaud Poterszman, Laszlo Tora, Jean-Marc Egly, Nicolas Le May, Frédéric Coin

**Affiliations:** 1 Institut de Génétique et de Biologie Moléculaire et Cellulaire Illkirch Cedex, C.U. Strasbourg, France; 2 Centre National de la Recherche Scientifique, UMR7104, 67404 Illkirch, France; 3 Institut National de la Santé et de la Recherche Médicale, U1258, 67404 Illkirch, France; 4 Université de Strasbourg, 67404 Illkirch, France; 5 Laboratoire Structure et Instabilité des Génomes, INSERM U1154, CNRS UMR7196, Muséum national d’Histoire naturelle, 43 rue Cuvier, 75005 Paris, France

## Abstract

The TFIIH subunit XPB is involved in combined Xeroderma Pigmentosum and Cockayne syndrome (XP-B/CS). Our analyses reveal that XPB interacts functionally with KAT2A, a histone acetyltransferase (HAT) that belongs to the hSAGA and hATAC complexes. XPB interacts with KAT2A-containing complexes on chromatin and an XP-B/CS mutation specifically elicits KAT2A-mediated large-scale chromatin decondensation. In XP-B/CS cells, the abnormal recruitment of TFIIH and KAT2A to chromatin causes inappropriate acetylation of histone H3K9, leading to aberrant formation of transcription initiation complexes on the promoters of several hundred genes and their subsequent overexpression. Significantly, this cascade of events is similarly sensitive to KAT2A HAT inhibition or to the rescue with wild-type XPB. In agreement, the XP-B/CS mutation increases KAT2A HAT activity in vitro. Our results unveil a tight connection between TFIIH and KAT2A that controls higher-order chromatin structure and gene expression and provide new insights into transcriptional misregulation in a cancer-prone DNA repair-deficient disorder.

## Abstract (web summary)

Chromatin structure plays a significant role in the regulation of gene expression. Here the authors show that TFIIH interacts with the histone acetyl transferase KAT2A and recruits the ATAC/hSAGA complexes to chromatin; and that loss of xeroderma pigmentosum group B (XPB) function results in chromatin decondensation and increased gene expression through activation of KAT2A.

## Introduction

The transcription factor IIH (TFIIH) is composed of ten subunits; XPB, p62, p52, p44, p34, and p8/TTDA which form the core complex, cdk7, MAT1, and cyclin H which form the cdk-activating kinase (CAK) sub-complex, linked to the core by XPD. In addition to its role as a basal transcription factor involved in RNA polymerase (Pol) II-dependent gene expression, TFIIH has also been implicated in nucleotide excision repair (NER)^1^. Inherited mutations in genes encoding three subunits of TFIIH lead to genetic disorders. Mutations in XPB trigger xeroderma pigmentosum (XP) combined with Cockayne syndrome (XP/CS) or trichothiodystrophy (TTD), mutations in XPD trigger XP alone, XP/CS or TTD and mutations in TTDA trigger only TTD^2–4^. These diseases have a broad spectrum of clinical features, including photosensitivity of the skin and high cancer predisposition mainly due to DNA repair deficiency and developmental and neurological defects likely related to transcriptional deregulation^5^. Consistent with the latter hypothesis, it has been shown in recent years that there are defects in several transcription activation pathways in XP/CS or TTD cells^5^.

XPB is a central TFIIH subunit that belongs to the SF2 helicase group, which is highly conserved in eukaryotes^6–8^. XPB has two highly conserved core RecA-like helicase domains (HD1 and HD2), which are found in all SF2 members^9^. Eukaryotic XPB also contains N- and C-terminal domains (NTD and CTD) that flank the central HD1 and HD2^6,10^. Interestingly, two of the three amino-acid substitutions in XPB found in XP/CS and TTD patients (F99S and T119P, respectively) are located in the NTD (from residues 1 to 320). XPB interacts with the p52 subunit of TFIIH through its NTD, resulting in an increase in its ATPase activity. The XP-B/CS F99S mutation weakens the XPB–p52 interaction and reduces anchoring of TFIIH to damaged DNA, which would explain the NER defect in related patients^11^. Although the NTD of XPB is clearly implicated in two rare genetic disorders, its role and the impact of XP-B/CS and TTD mutations on its function have been insufficiently studied.

To better understand the role of the NTD of XPB and the impact of human XPB mutations on cellular homeostasis, we tethered several XPB mutants to chromatin using the lacO/LacR reporter system^12,13^, and analyzed chromatin structure using confocal microscopy and three-dimensional (3-D) reconstruction of the cell nucleus. We first showed that the deletion of XPB NTD induces large chromatin decondensation. We then demonstrated that the XP-B/CS mutation (F99S) mimicks the deletion of the NTD by inducing a similar chromatin decondensation, but the TTD mutation (T119D) does not. In order to address the mechanisms, we demonstrated that TFIIH/XPB interacts with KAT2A (GCN5), a histone acetyltransferase (HAT) that is a subunit of the Spt Ada Gcn5 acetyltransferase (hSAGA) and Ada two A-containing (hATAC) complexes^14–16^. Using an in vitro histone acetyltransferase assay, we observed that TFIIH-XPB^F99S^ strongly enhances the enzymatic activity of KAT2A. Cells derived from the corresponding XP-B/CS patient have a global increase in H3K9 acetylation and a decrease of H3K9 methylation that trigger overexpression of several hundred genes. We further showed that co-recruitment of TFIIH-XPB^F99S^ and KAT2A on chromatin results in the accumulation of the H3K9ac mark and the formation of Pol II initiation complexes at the promoters of overexpressed genes. We were able to restore the chromatin state, the promoter occupancy and the transcription program by expressing wild-type XPB or by inhibiting KAT2A HAT activity, highlighting the close relationship that exists between these two fundamental cellular actors.

## Results

### Tethered XPB mutants induce large chromatin decondensation

**Fig. 1:**
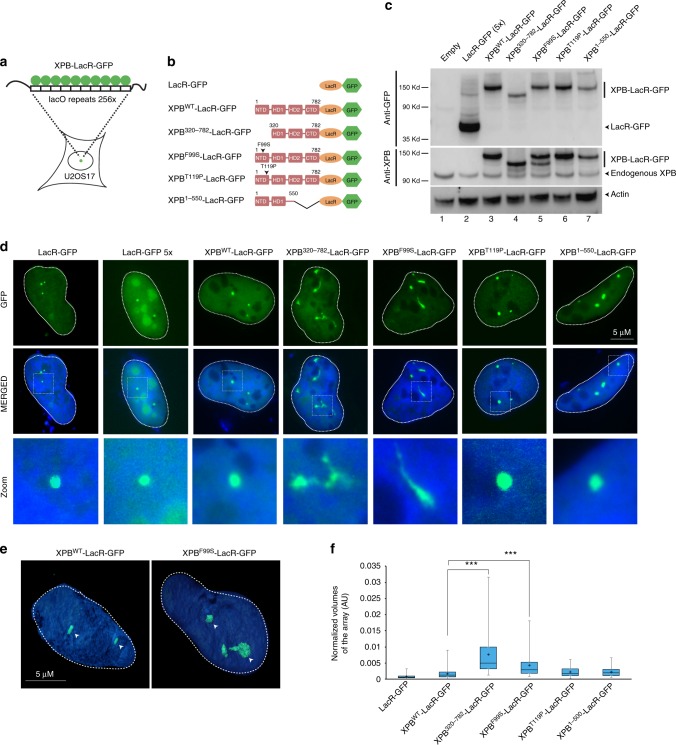
A loss of XPB NTD integrity induces large-scale chromatine decondensation. **a** Schematic representation of the lacO/LacR tethering system used in U2OS17 cells. See the Materials and methods section for a full description of the cell line. **b** Schematic representation of wild-type and mutant XPB-LacR-GFP constructs. For clarity, the sizes of the GFP (238 aa) and LacR (367 aa) are omitted. **c** Proteins from whole-cell extracts (15 μg) of U2OS17 cells transiently transfected with wild-type or mutant XPB-LacR-GFP constructs were resolved by SDS-PAGE and immunoblotted using either polyclonal rabbit anti-GFP (upper panel), polyclonal rabbit anti-XPB (middle panel) or monoclonal mouse anti-Actin antibodies (lower panel). Source data are provided as a Source Data file. **d** U2OS17 cells were transiently transfected with 1 μg of expression vectors for the following proteins: LacR-GFP, XPB^WT^-LacR-GFP, XPB^320–782^-LacR-GFP, XPB^F99S^-LacR-GFP, XPB^T119P^-LacR-GFP, XPB^1–550^-LacR-GFP. In parallel, U2OS17 cell line was transiently transfected with 5 μg of expression vector for LacR-GFP. GFP was observed by fluorescence microscopy 24 h post transfection. Lower panels are magnifications of the white rectangles in the upper panels. **e** 3D reconstruction of U2OS17 cellular nuclei transiently transfected either with XPB^WT^-LacR-GFP or with XPB^F99S^-LacR-GFP using Imaris Software (Bitplane). **f** Whisker box plot shows quantifications of the relative array volumes (volume of the array/volume of the nucleus; 40–150 cells for each condition). Significant *p*-values are indicated (*** ≤ 0.001) and were obtained using a Kruskal Wallis test. Source data are provided as a Source Data file

To directly assess the impact of XPB NTD on chromatin structure and organization, we first used the lac operator-repressor (lacO-LacR) tethering system^12,13^. Constructs that express the lac repressor DNA binding domain (LacR) fused in frame to XPB and GFP were transfected into the human U2OS17 cells that have repetitive binding sites for lacO integrated in the genome^17^ (Fig. 1a). GFP facilitates monitoring the proteins on chromatin. Given the implication of XPB NTD in human disease, we tested three XPB NTD mutants that include a complete NTD deletion (XPB^320–782^), a substitution (XPB^F99S^) expressed in XP-B/CS patient-derived cells (XPCS1BA = XP-B/CS^F99S^) and a substitution (XPB^T119P^) expressed in TTD patient-derived cells (TTD6VI = TTD-XPB^T119P^). We also tested a large truncation of the C-terminus that deletes the CTD and HD2 (XPB^1–550^) domains (see XPB^WT^-LacR-GFP, XPB^320–782^-LacR-GFP, XPB^F99S^-LacR-GFP, XPB^T119P^-LacR-GFP, and XPB^1–550^-LacR-GFP; Fig. 1b, c; the western blot shows that the constructs express expected levels of appropriately-sized proteins).

Expression of LacR-GFP in U2OS17 cells^13,18^ marked the lacO repeat clusters with small condensed dots (Fig. 1d). We did not observe these “GFP” spots in the parental U2OS cells that do not have the LacO repeat cluster and that were transfected under the same conditions^13^. After transient transfection of the XPB-fused constructs, we observed that tethering of the NTD deletion mutant XPB^320–782^-LacR-GFP to the lacO arrays caused the transformation of the small condensed dots into unshaped and fiber-like structures, whereas tethering of wild-type XPB (XPB^WT^-LacR-GFP construct) did not induce this decondensation (Fig. 1d). Interestingly, the XP-B/CS mutant also induced chromatin decondensation that was comparable to that exhibited by the NTD deleted mutant (Fig. 1d). In contrast, array decondensation was not observed after the tethering of the TTD point or CTD deletion mutants (Fig. 1d). To rule out that overexpression of the LacR repressor alone was responsible for array decondensation, we used a higher transfection level of LacR-GFP (corresponding to 5× of the amount transfected above) (Fig. 1c) and observed no opening of the arrays in these conditions (Fig. 1d). More than 80% of the transiently transfected cells showed decondensation of at least one lacO array/cell in U2OS17 cells transfected with XPB^F99S^-LacR-GFP or XPB^320–782^-LacR-GFP, while no decondensation was observed with LacR-GFP at low or high transfection levels (Supplementary Fig. 1a). In addition, low level expression of XPB^F99S^-LacR-GFP in stably transfected U2OS17 cells still induced consistent chromatin decondensation compared to XPB^WT^-LacR-GFP (Supplementary Fig. 1b). Finally, robust unfolding of chromatin fibers was also observed after transient transfection of XPB^320–782^-LacR-GFP and XPB^F99S^-LacR-GFP into the A0-3 hamster reporter cell line^12^ (Supplementary Fig. 1c), suggesting that XPB^F99S^ and XPB^320–782^-mediated decondensation is a general phenomenon.

To quantify the size of the LacR-bound arrays, we used rapid confocal microscopy and 3-D reconstruction of the images (Fig. 1e). The images were taken 24 h after transfection of the LacR-GFP constructs and the volume filled with the GFP signal in 3-D was measured and normalized by the volume of the nucleus in 3-D. We found an average fivefold increase in the size of the array following the tethering of XPB^320–782^ or XPB^F99S^ mutants compared to XPB^WT^ (Fig. 1f). ATP depletion had no impact on XPB^F99S^-induced chromatin decondensation while it suppressed unfolding of the array upon the tethering of the chromatin remodeler DDB2 used as control (Supplementary Fig. 1d)^19^. Altogether, these observations demonstrate that prolonged binding of the XPB^320–782^ or XPB^F99S^ mutants to chromatin triggers large-scale chromatin decondensation that is independent of ATP.

### The XP/CS mutation increases deposition of H3K9ac mark

**Fig. 2:**
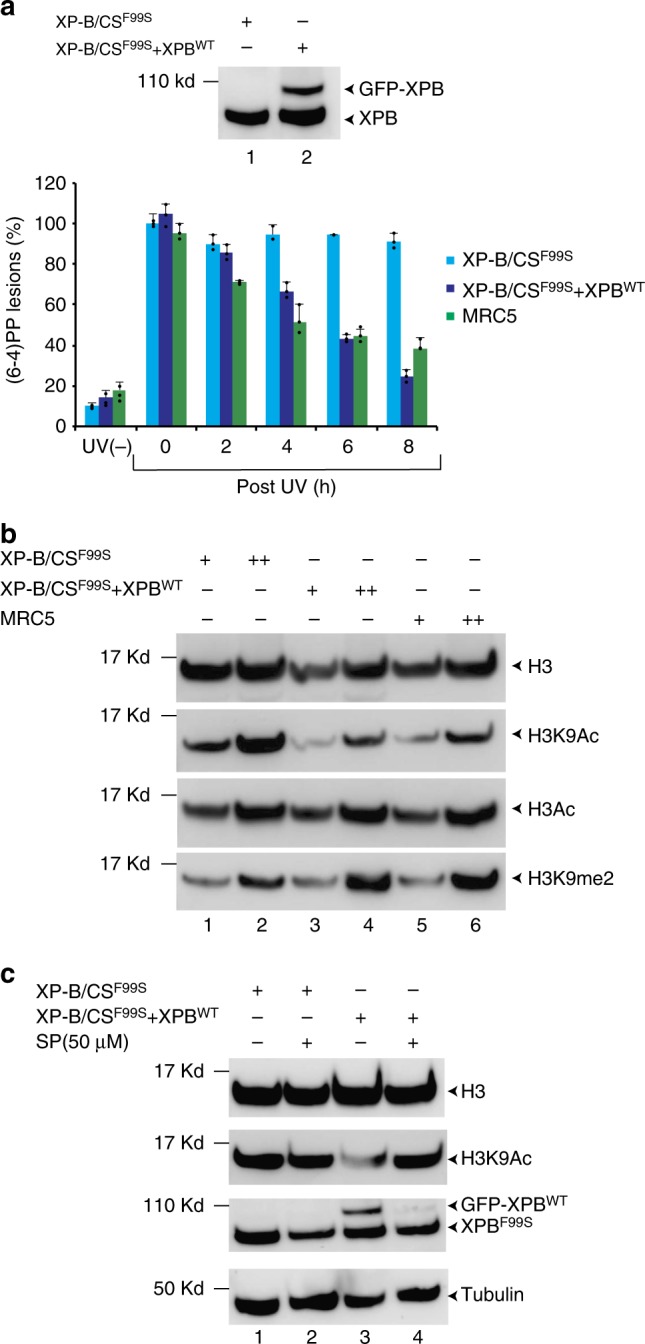
XP-B/CS^F99S^ patient-derived cells have a global increase in the H3K9ac histone mark. **a** Extracts from either XP-B/CS^F99S^ or XP-B/CS^F99S^ + XPB^WT^ cells were resolved by SDS-PAGE and immunoblotted with a monoclonal mouse anti-XPB antibody. Source data are provided as a Source Data file. (6-4)PP removal measurements were carried out in XP-B/CS^F99S^, stably transfected XP-B/CS^F99S^ + XPB^WT^ and wild-type MRC5 cells harvested at different time points after UV irradiation at 30 J/m^2^ as indicated. Cells were labeled with a monoclonal mouse anti-(6-4)PP antibody and signals were measured using a INCell 1000 analyzer (GE Healthcare). The graph represents the percentage of lesions remaining in the genome at a given time (error bars represent SD from three independent experiments). For each time point, about 20000–40000 cells were analyzed. **b** Histones were extracted from either XP-B/CS^F99S^, XP-B/CS^F99S^ + XPB^WT^ or wild-type MRC5 fibroblasts, resolved by SDS-PAGE and immunoblotted with either polyclonal rabbit anti-histone H3, polyclonal rabbit anti-histone H3ac, monoclonal mouse anti-histone H3K9ac or polyclonal rabbit anti-histone H3K9me2. Source data are provided as a Source Data file. **c** XP-B/CS^F99S^ or XP-B/CS^F99S^ + XPB^WT^ cells were treated 3 h with DMSO or SP (50 μM) and histones were extracted, resolved by SDS-PAGE and immunoblotted either with a polyclonal rabbit anti-histone H3 or with monoclonal mouse anti-histone H3K9ac antibodies. In parallel, cell extracts were resolved by SDS-PAGE and western blotted with either a monoclonal mouse anti-XPB or polyclonal rabbit anti-tubulin antibodies. Source data are provided as a Source Data file

Thus far our results indicate that the XP/CS mutation localized in the XPB NTD specifically induces large-scale chromatin decondensation. We then set out to analyze the impact of this mutation in patient-derived XPCS1BA cells (XP-B/CS^F99S^)^20^ that we compared to XPCS1BA cells expressing wild-type GFP-tagged XPB (XP-B/CS^F99S^ + XPB^WT^)^21^ (Fig. 2a, upper panel). To validate this cell line, we analyzed its DNA repair activity and observed that the GFP-XPB^WT^ expressing cells removed (6-4)PP lesions with kinetics similar to that of the control MRC5 wild-type fibroblasts (Fig. 2a, lower panel) indicating that XP-B/CS^F99S^ + XPB^WT^ had regained wild-type levels of DNA repair activity. We conclude from these experiments that the exogenously expressed XPB-GFP is functional as previously demonstrated^21^.

Using these cell lines, we next analyzed several H3 modifications and observed a higher level of global H3K9ac in XP-B/CS^F99S^ cells compared to GFP-XPB^WT^ expressing cells or to wild-type MRC5 fibroblasts (Fig. 2b). In contrast, the global level of H3 acetylation was similar in the three cell lines. H3K9 residue is either acetylated or methylated, two modifications that are mutually exclusive^22^. Accordingly, we observed that increased acetylation of H3K9 in XP-B/CS^F99S^ was accompanied by a decrease in H3K9 di-methylation (me2), compared to stably transfected GFP-XPB^WT^ expressing cells or wild-type fibroblasts (Fig. 2b). In contrast, the levels of several other modifications, such as H3K4me3, H3K14ac or H3S10 phosphorylation were similar (Supplementary Fig. 2).

To further confirm that re-expression of XPB^WT^ in the stably transfected cells was responsible for decreasing the level of H3K9ac, we treated cells for 3 h with Spironolactone (SP), a small molecule that induces rapid degradation of XPB^23^. Interestingly, XPB^F99S^ was more resistant than XPB^WT^ to SP treatment, and only the amount of XPB^WT^ decreased dramatically in XP-B/CS^F99S^ + XPB^WT^ cells (Fig. 2c). Therefore, SP treatment quickly restored the original XP-B/CS^F99S^ context in these cells. After 3 h of SP treatment, the rapid degradation of XPB^WT^ increased the level of H3K9ac (compare lanes 3 and 4) that reached the level observed in the parental XP-B/CS^F99S^ cells (compare lanes 4 and 1, 2). All together, these data demonstrate that XPB^F99S^ impacts posttranslational modifications of histones and chromatin structure in cells derived from the XP-B/CS^F99S^ patient.

### TFIIH recruits KAT2A-containing HAT complexes to chromatin

**Fig. 3:**
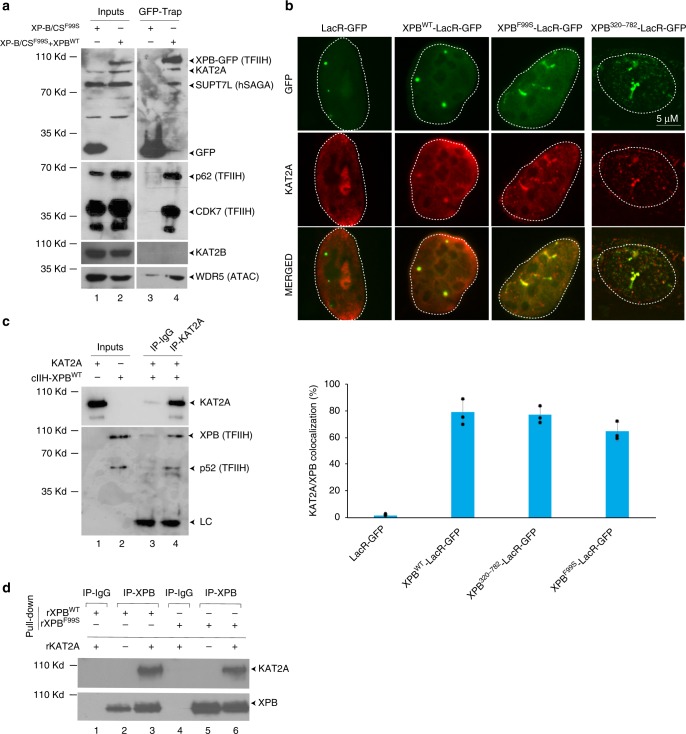
XPB recruits KAT2A-containing HAT complexes to chromatin. **a** TFIIH-XPB^WT^ was immunoprecipitated from nuclear extracts of stably transfected cells using anti-GFP antibodies (GFP-Trap) and washed with 200 mM salt (Lanes 2 and 4). Control IPs were performed with anti-GFP on an XP-B/CS^F99S^ cell line stably expressing GFP alone (Lanes 1 and 3). Proteins on the resin were resolved by SDS-PAGE and immunoblotted using polyclonal rabbit anti-GFP, monoclonal mouse anti-p62, polyclonal rabbit anti-CDK7, polyclonal rabbit anti-KAT2A (SCBT) (Supplementary Figure 9), polyclonal rabbit anti-KAT2B, polyclonal rabbit anti-WDR5, or polyclonal rabbit anti-SUPT7L antibodies. Source data are provided as a Source Data file. **b** The U2OS17 cell line was transiently transfected with 1 μg of expression vectors for the following proteins: LacR-GFP, XPB^WT^-LacR-GFP, XPB^F99S^-LacR-GFP, or XPB^320–782^-LacR-GFP together with 1 μg of expression vector for Flag-KAT2A. Colocalization of KAT2A with GFP was detected by immunofluorescence staining using a polyclonal rabbit anti-flag antibody. The values on the graph represent the percentage of colocalization of KAT2A with GFP on the array (error bars represent SD from three independent quantifications). **c** Purified wild-type recombinant core TFIIH (cIIH-XPB^WT^) was incubated with purified Flag-KAT2A (400 ng) and pull-down assays were performed using either unspecific anti-IgG or polyclonal rabbit anti-KAT2A (SCBT) antibody (Supplementary Figure 9). After washing, proteins on the resin were resolved by SDS-PAGE and immunoblotted using mouse monoclonal anti-XPB or anti-p52 antibodies (two subunits of TFIIH) or monoclonal mouse anti-KAT2A (IGBMC) antibody. Source data are provided as a Source Data file. **d** Bacterially expressed recombinant KAT2A (rKAT2A) (400 ng) was tested for its ability to interact with either rXPB^WT^ (lanes 1–3) or rXPB^F99S^ (lanes 4–6) (500 ng) in a pulled-down assay (IP-XPB). After washing, proteins on the resin were resolved by SDS-PAGE and immunoblotted using mouse monoclonal anti-XPB and mouse monoclonal anti-KAT2A antibodies (IGBMC). Lanes 1 and 4, immunoprecipitations with an irrelevant antibody (IP-IgG). Source data are provided as a Source Data file

Acetylation of H3K9 is mainly performed by the histone acetyl transferases KAT2A (GCN5) and KAT2B (PCAF)^24^, which are mutually exclusive subunits of the hATAC or hSAGA complexes^16^. We used GFP-Trap^®^ to immunoprecipitate exogenous GFP-tagged XPB^WT^ from nuclear extracts of XP-B/CS^F99S^ + XPB^WT^ cells. We observed that KAT2A, SUPT7L (a subunit of hSAGA) and WDR5 (a subunit of hATAC) were pulled-down with XPB^WT^, as well as additional subunits of the TFIIH complex (Fig. 3a). Interestingly, we did not observe co-immunoprecipitation of KAT2B (Fig. 3a), suggesting that TFIIH interacts with the hSAGA/hATAC complexes through KAT2A. To demonstrate that endogenous TFIIH interacts with KAT2A-containing complexes, we also used a CRISPR/Cas9 engineered U2OS cell line harboring endogenous homozygous XPB-fused C-terminally to GFP (U2OS^XPB::GFP^) (Supplementary Fig. 3a). Using GFP-trap, we observed co-immunoprecipitation of KAT2A, ZZZ3 (hATAC), and SUPT7L (hSAGA) together with XPB and p44 from U2OS^XPB::GFP^ cells (Supplementary Fig. 3b).

We then co-transfected U2OS17 reporter cells with plasmids encoding Flag-KAT2A and either LacR-GFP, XPB^WT^-LacR-GFP, XPB^F99S^-LacR-GFP, or XPB^320–782^-LacR-GFP. We observed colocalization of KAT2A with the XPB^WT^ and XPB^F99S^ constructs on chromatin, which was not detected with LacR-GFP (Fig. 3b). Interestingly, KAT2A also co-localized with the NTD deletion mutant XPB^320–782^-LacR-GFP (Fig. 3b). In addition, we purified recombinant wild-type core TFIIH (cIIH-XPB^WT^) expressed in insect cells^11^ and observed direct interaction with recombinant KAT2A (rKAT2A) in vitro (Fig. 3c). Pull-down experiments further demonstrated that rKAT2A interacts directly with both rXPB^WT^ and rXPB^F99S^ (Fig. 3d). Together, these data indicate that TFIIH interacts with both KAT2A-containing hSAGA and hATAC complexes and that F99S mutations or NTD deletion do not alter these interactions.

### Loss of XPB NTD integrity increases KAT2A HAT activity

**Fig. 4:**
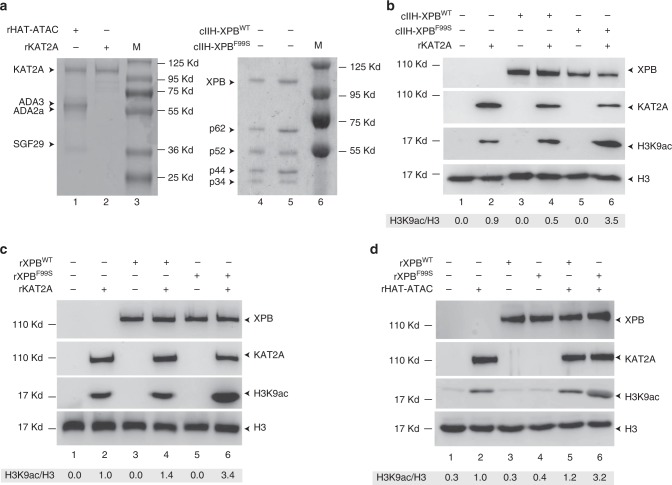
Loss of XPB NTD integrity induces an increase in KAT2A HAT activity. **a** rKAT2A and the recombinant HAT-ATAC module (rHAT-ATAC) containing KAT2A, ADA3, ADA2a, and SGF29 were resolved by SDS-PAGE followed by Coomassie staining. Core TFIIH containing p62, p52, p44, p34 and either XPB^WT^ (cIIH-XPB^WT^) or XPB^F99S^ (cIIH-XPB^F99S^) were resolved by SDS-PAGE followed by Coomassie staining. Source data are provided as a Source Data file. **b** One hundred nanograms of core TFIIH containing either XPB^WT^ (cIIH-XPB^WT^) or XPB^F99S^ (cIIH-XPB^F99S^) were incubated with 50 ng rKAT2A together with histone H3.3 and cold acetyl-CoA. Following resolution by SDS-PAGE, proteins were immunoblotted using polyclonal rabbit anti-H3, monoclonal mouse anti-H3K9Ac, mouse monoclonal anti-XPB, and polyclonal rabbit anti-KAT2A (SCBT) antibodies. Quantification of H3K9Ac was performed using ImageJ software and normalized with H3. Source data are provided as a Source Data file. **c** Twenty nanograms of rXPB^WT^ or rXPB^F99S^ were incubated with 50 ng of rKAT2A together with histone H3.3 and cold acetyl-CoA. Following incubation, reactions were treated as described in panel (**b**). Source data are provided as a Source Data file. **d** Twenty nanograms of rXPB^WT^ or rXPB^F99S^ were incubated with 200 ng of the rHAT-ATAC module together with histone H3.3 and cold Acetyl-CoA. Following incubation, reactions were treated as described in panel (**b**). Source data are provided as a Source Data file

To test whether the enzymatic activity of KAT2A could be modified by TFIIH, we performed in vitro HAT assays^25^. We incubated purified recombinant wild type or F99S core TFIIH (cIIH-XPB^WT^ or cIIH-XPB^F99S^)^11^ (Fig. 4a, lanes 4, 5) with purified recombinant KAT2A (rKAT2A) (lane 2)^25^ and unmodified histone H3.3 in the presence of cold acetyl-CoA (acetyl donor). After incubation with cIIH-XPB^F99S^, we observed a strong increase in KAT2A HAT activity that was not detected with cIIH-XPB^WT^ (Fig. 4b). To investigate whether XPB alone is sufficient, we performed HAT assays with recombinant XPB^11^ and demonstrated that rXPB^F99S^ increases the HAT activity of rKAT2A (Fig. 4c). To test the effect of other components of the HAT complex, we produced the recombinant human HAT module of the ATAC complex (rHAT-ATAC) that contains the KAT2A, ADA2a, ADA3, and SGF29 subunits^25^ (Fig. 4a, lane 1) and demonstrated that rXPB^F99S^ also increases the HAT activity of the complex (Fig. 4d).

**Fig. 5:**
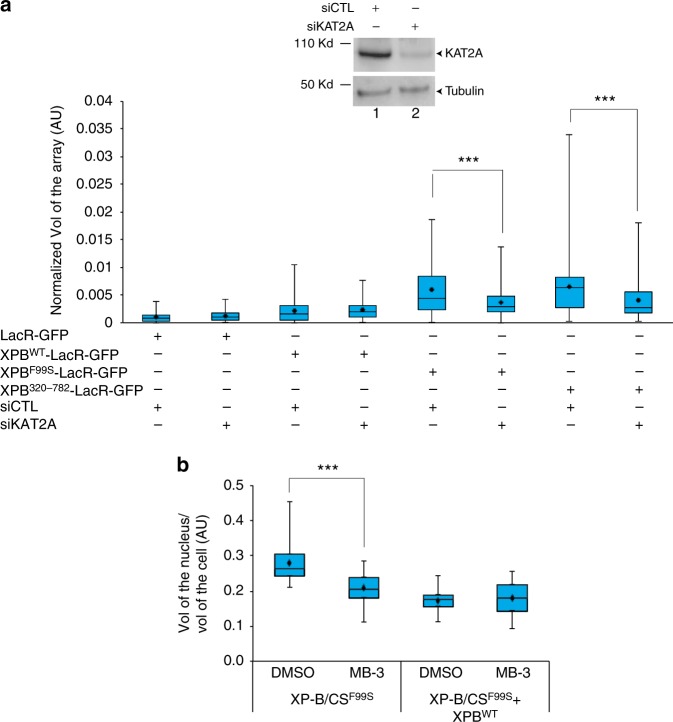
Higher-order chromatin decondensation induced by XPB mutants is due to KAT2A HAT activity. **a** U2OS17 cells were transfected either with siCTL or with siKAT2A for 72 h and cell extracts were resolved by SDS-PAGE and immunoblotted using polyclonal rabbit anti-KAT2A or polyclonal rabbit anti-tubulin antibodies. siCTL or siKAT2A transfected cells were re-transfected with either LacR-GFP, XPB^WT^-LacR-GFP, XPB^F99S^-LacR-GFP or XPB^320–782^-LacR-GFP for 24 h and the relative array volumes (Vol) were quantified as described above. Significant *p*-value are indicated (*** ≤ 0.001) and were obtained using a Kruskal Wallis test. Source data are provided as a Source Data file. **b** XP-B/CS^F99S^ and XP-B/CS^F99S^ + XPB^WT^ cells were treated or not with MB-3 (200 μM) for 15 h and were subsequently labeled with polyclonal rabbit anti-tubulin and stained with DAPI. Cells were then reconstructed in 3D using Imaris Software (Bitplane) and the volumes of the cells and their nuclei were measured. The whisker box plot shows the volume of the nucleus/volume (Vol) of the cell (at least 30 cells for each condition, error bars represent SD from three independent experiments). The significant *p*-value is indicated (*** ≤ 0.001) and were obtained using a Kruskal Wallis test. Source data are provided as a Source Data file

We then depleted KAT2A in U2OS17 cells using small interfering (si)RNA and detected a significant decrease in chromatin decondensation induced by the tethering of XPB^F99S^ or XPB^320–782^, whereas no modifications were detected upon tethering of XPB^WT^ (Fig. 5a).

We also tested whether chromatin decondensation in XP-B/CS^F99S^ was large enough to alter the size of the nucleus. We observed that the XPB^F99S^ mutation induces a twofold increase in the size of XP-B/CS^F99S^ nuclei compared to XP-B/CS^F99S^ + XPB^WT^ cells (Fig. 5b). The increase in the size of the XP-B/CS^F99S^ nucleus was reduced by the addition of inhibitor of KAT2A, MB-3 (Butyrolactone 3)^26^, arguing that it is directly linked to KAT2A activity (Fig. 5b). Altogether, these data suggest that loss of integrity in XPB NTD induces an increase in KAT2A-containing HAT complex activity triggering large aberrant chromatin decondensation.

### KAT2A induces gene activation in XP-B/CS cells

**Fig. 6:**
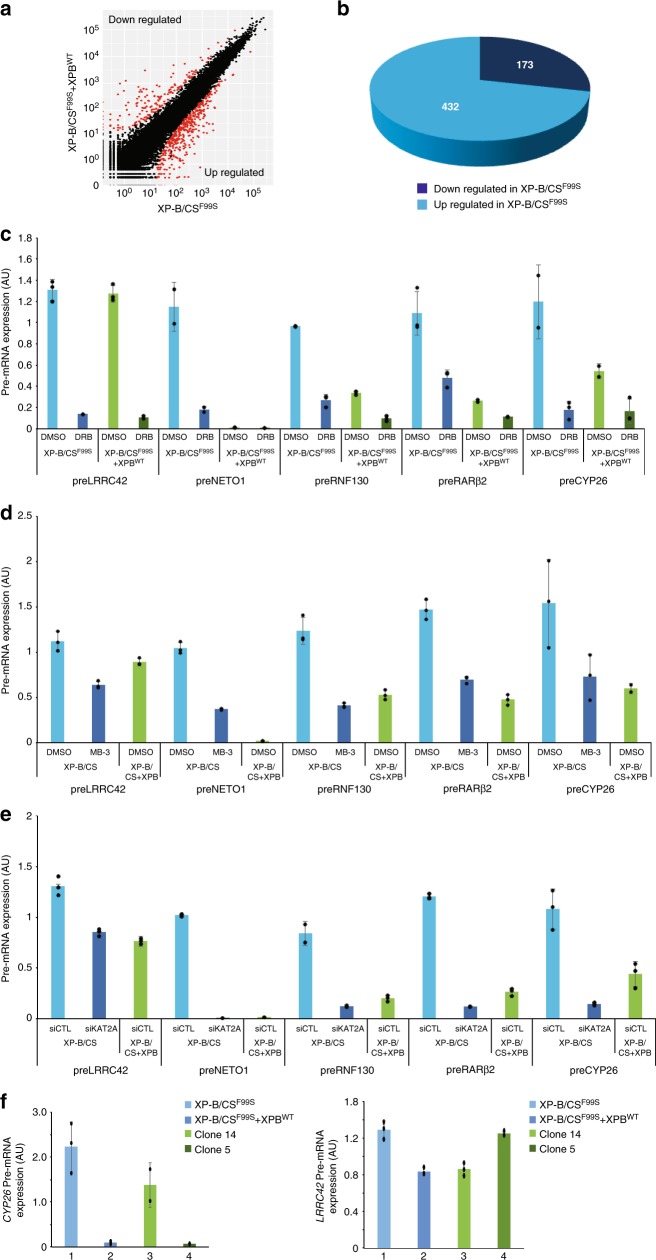
KAT2A HAT activity induces inappropriate gene activation in XP-B/CS^F99S^ cells. **a** RNA-seq analysis scatter plots comparing XP-B/CS^F99S^ vs XP-B/CS^F99S^ + XPB^WT^ transcription profiles. Points show significantly over- (bottom) or under-(top) represented mRNA in XP-B/CS^F99S^ cells compared to XP-B/CS^F99S^ + XPB^WT^. All data were evaluated with the DESeq2 R package. For a given gene, its value is the normalized gene expression value relative to the mean of all samples belonging to the same condition. **b** A Comparative analysis of RNA-seq data from XP-B/CS^F99S^ and XP-B/CS^F99S^ + XPB^WT^. The pie chart indicates the number of genes that are up- and downregulated in the XP-B/CS^F99S^ vs XP-B/CS^F99S^ + XPB^WT^. **c** Pre-mRNA levels (error bars represent SD from three independent experiments) of *LRRC42, NETO1, RNF130*, *RARβ2*, and *CYP26* were analyzed in XP-B/CS^F99S^ or XP-B/CS^F99S^ + XPB^WT^ cells treated with either DMSO or DRB, as indicated. Data represent the relative expression levels of the pre-mRNA vs. GAPDH mRNA. Source data are provided as a Source Data file. **d** Pre-mRNA levels (error bars represent SD from three independent experiments) of *LRRC42, NETO1, RNF130*, *RARβ2*, and *CYP26* were analyzed in XP-B/CS^F99S^ or XP-B/CS^F99S^ + XPB^WT^ cells treated either with DMSO or with MB-3 (200 μM) as indicated. Data represent the relative expression levels of the pre-mRNA vs. GAPDH mRNA. Source data are provided as a Source Data file. **e** Pre-mRNA levels (error bars represent SD from three independent experiments) of *LRRC42, NETO1, RNF130*, *RARβ2*, and *CYP26* were analyzed in XP-B/CS^F99S^ or XP-B/CS^F99S^ + XPB^WT^ cells treated with either siCTL or siKAT2A, as indicated. Data represent the relative expression levels of the pre-mRNA vs. GAPDH mRNA. Source data are provided as a Source Data file. **f** Pre-mRNA levels (error bars represent SD from three independent experiments) of *CYP26* and *LRRC42* were analyzed in XP-B/CS^F99S^ and XP-B/CS^F99S^ + XPB^WT^ cells as well as on stable clone 14 and clone 5 that were selected after transfection of cDNA coding for XPB^T119P^. Clone 14 does not express the transgene while clone 5 expresses only the transgene. Data represent the relative expression levels of the pre-mRNA vs. GAPDH mRNA. Source data are provided as a Source Data file

We then sought to analyze the biological significance of our findings and analyzed the transcriptomic profiles by RNA sequencing of cells derived from the XP-B/CS^F99S^ patient. We observed an increased amount of 432 mRNAs in the patient’s cells, compared to stably transfected XP-B/CS + XPB^WT^ cells (Fig. 6a, b and Supplementary Data 1).

To confirm these results, we analyzed the accumulation of pre-mRNA using intron/exon junction amplification by RT-qPCR to evaluate the amount of newly synthetized mRNA. The pre-mRNA accumulation of five representative genes were studied, including *LRRC42*, whose expression was similar in the two cell lines, and *NETO1*, *RNF130*, *RARβ2*, and *CYP26*, that were upregulated in XP-B/CS^F99S^ cells. Their pre-mRNA levels were rapidly inhibited by 1h treatment with the inhibitor of class II gene expression 5,6-dichloro-1-β-D-ribofuranosylbenzimidazole (DRB) (Fig. 6c), indicating that we had amplified pre-mRNA. While *LRRC42* pre-mRNA accumulation was similar in both cell lines, we detected a higher amount of *NETO1*, *RNF130*, *RARβ2,* and *CYP26* pre-mRNA in DMSO-treated patient cells compared to DMSO-treated stably transfected cells (Fig. 6c), implying that these genes were more transcribed in the XP-B/CS^F99S^ patient-derived cells. When we treated the patient’s cells with the inhibitor of KAT2A, MB-3 or with siKAT2A, we observed a significant repression of pre-mRNA expression *for NETO1*, *RNF130*, *RARβ2*, and *CYP26*, which dropped to the levels observed in the stably transfected cells (Fig. 6d, e). In contrast, the expression *of LRRC42* was only moderately affected after treatment with MB-3 or siKAT2A (Fig. 6d, e).

In order to analyze whether the XPB^F99S^ mutation was responsible for the overexpression of the representative genes that we selected to study (see above), we used a model system developed by Dr. Sarasin that can be used to correlate the relative expression levels of XPB^F99S^ (XP/CS) and XPB^T119P^ (TTD) with gene expression profiles^27^. In this system, the patient cell line XP-B/CS^F99S^ was originally transfected with a plasmid expressing XPB^T119P^ and two clones were isolated; clone 14 does not express the ectopic XPB^T119P^ and therefore only expressed the endogenous XPB^F99S^ while clone 5 only express the ectopic XPB^T119P^ ^27^. We observed a decrease in the expression of the representative *CYP26* gene with the expression of the mutant XPB^T119P^ (compare clones 14 and 5; Fig. 6f, left panel) while the expression of *LRCC42* was similar in the two clones (Fig. 6f, right panel). These data indicate, first, that XP/CS-XPB^F99S^ is responsible for the overexpression of genes observed in the patient-derived cells and, second, that the mutant TTD-XPB^T119P^, which does not have an impact on chromatin structure (see Fig. 1), does not alter the expression of the representative genes.

### Transcription repression recovery in XP-B/CS cells

**Fig. 7:**
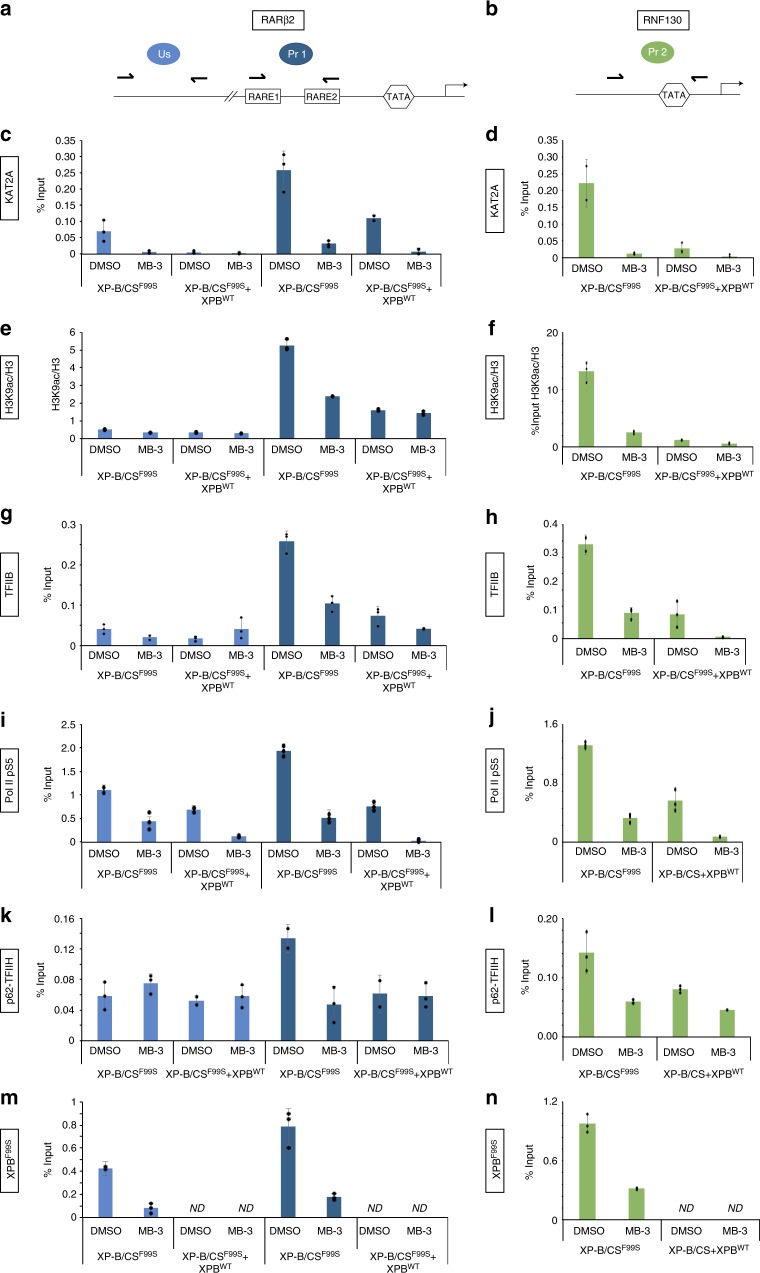
Chromatin modification and RNA Pol II TIC formation at the promoters of overexpressed genes in XP-B/CS cells. **a**, **b** Schematic representations of the *RARβ2* (**a**) and *RNF130* (**b**) gene regions, including the upstream (Us) and promoter (Pr 1 and 2, respectively) regions that were amplified (see in facing half-arrowheads). **c**–**j** ChIPs monitoring occupancy by KAT2A (using a polyclonal rabbit anti-KAT2A antibody (EpiGentek) Supplementary Figure 9) (**c**, **d**), H3K9ac/H3 (using monoclonal mouse anti-H3K9Ac and polyclonal rabbit anti-H3 antibodies) (**e**, **f**), TFIIB (using a polyclonal rabbit anti-TFIIB antibody) (**g**, **h**) and Pol II pS5 (using a monoclonal rat anti-Pol II pS5 antibody) (**i**, **j**) of either the Us or the Pr regions of *RARβ2* (**c**, **e**, **g**, **i**) or the Pr region of *RNF130* (**d**, **f**, **h**, **j**) in XP-B/CS^F99S^ and XP-B/CS^F99S^ + XPB^WT^ cells treated either with DMSO or with MB-3 (200 μM). **k**–**n** ChIPs monitoring occupancy by the TFIIH core subunits p62 (using polyclonal rabbit anti-p62 antibody) (**k**, **l**) and XPB^F99S^ (using polyclonal rabbit anti-XPB antibody) (**m**, **n**) of the Us or Pr regions of *RARβ2* (**k**–**m**) or of the Pr region of *RNF130* (**l**–**n**) in XP-B/CS^F99S^ treated with either DMSO or MB-3

We performed chromatin immunoprecipitation (ChIP) in XP-B/CS^F99S^ and XP-B/CS^F99S^ + XPB^WT^ cells and monitored the deposition of histone modification marks and the formation of the RNA Pol II transcription-initiation complex (TIC) on the promoters of the representative gene (Fig. 7a, b). In XP-B/CS^F99S^ cells, we observed a concordance between KAT2A recruitment and the deposition of the H3K9ac mark on the *RARβ2*, *RNF130*, *CYP26*, and *NETO1* promoters (Fig. 7c–f and Supplementary Fig. 4a, b). In parallel, we also performed ChIPs deposition of the H3K9ac mark on a non-coding region located 130 kb upstream of the *RARβ2* promoter (Fig. 7a)^28^ and observed a slight increase in KAT2A recruitment and H3K9 acetylation in this region, but to a lesser extent than on the *RARβ2* promoter (Fig. 7c, e). The subsequent events, the recruitment of both the basal transcription factor TFIIB (Fig. 7g, h and Supplementary Fig. 4c) and the initiation form of RNA Pol II phosphorylated on Serine 5 (Pol II pS5) (Fig. 7i–j and Supplementary Fig. 4d) were observed on the promoters of overexpressed genes. Intriguingly, we observed that either MB-3 treatment or expression of XPB^WT^ equally led to elimination of KAT2A, decreases in the deposition of H3K9ac and eviction of TFIIB and RNA Pol II pS5 from these promoters (Fig. 7c–j and Supplementary Fig. 4a–d).

We then analyzed the recruitment of two TFIIH subunits to the overexpressed promoter models. First, we observed that the p62 subunit of the core TFIIH was recruited at these promoters only in DMSO-treated XP-B/CS^F99S^ cells (Fig. 7k, l and Supplementary Fig. 4e). Treatment with MB-3 or expression of XPB^WT^ equally led to the eviction of the core TFIIH subunit from these promoters (Fig. 7k, l and Supplementary Fig. 4e). Second, we analyzed the deposition of the XPB^F99S^ mutant in XP-B/CS^F99S^ cells. Interestingly, accumulation of XPB^F99S^ on overexpressed promoters was removed after treatment with MB-3 (Fig. 7m, n and Supplementary Fig. 4f).

Finally, we analyzed the promoter of the *LRRC42* gene and noted a slight increase in H3K9ac in XP-B/CS^F99S^ compared to XP-B/CS^F99S^ + XPB^WT^ cells that was sensitive to MB-3 treatment (Supplementary Fig. 5a). However, and in good agreement with the similar expression of *LRRC42* in the patient and the derived stably transfected cells, ChIP indicated a similar enrichment of TFIIB and Pol II S5 on the promoters of *LRRC42* in these cell lines, which was insensitive to MB-3 treatment or to the re-introduction of XPB^WT^ (Supplementary Fig. 5b, c).

Together, these data reveal strong recruitment of TFIIH^F99S^ and KAT2A to specific promoters in patient cells, leading to the formation of RNA Pol II TIC and gene overexpression. This cascade of events is equally sensitive to KAT2A inhibition or to rescue with XPB^WT^.

## Discussion

The findings of the current study reveal a close connection between two fundamental cellular proteins, XPB and KAT2A. A global increase in H3K9ac mark deposition, unfolding of higher level chromatin structure and deregulation of gene expression appear to be mediated through an increase in KAT2A HAT activity induced by an XP/CS mutation in XPB.

Although artificial, the lacO/LacR system provides a very fast and powerful assay for analyzing the impact of chromatin-binding factors on higher-order chromatin structure in vivo^12,19,29,30^. However, it is often difficult to know if the proteins inducing chromatin decondensation in the lacO/LacR system are involved in large-scale chromatin remodeling in a normal environment or only in local changes to histone modifications. Robust and consistent unfolding of chromatin fibers was also observed in the A0-3 hamster reporter cell line, suggesting that XPB^F99S^ and XPB^320–782^-mediated decondensation is a general phenomenon. In comparison, we did not detect chromatin decondensation when we tethered the acidic activation domain of the transcription activator VP16 in U20S17 (Supplementary Fig. 6) contrary to the KAT2A-dependent large-scale chromatin changes that it induced in A0-3^12^. These observations suggest that mutated XPB induces remodeling of chromatin that differs mechanistically from local chromatin changes observed following the tethering of a transcriptional activator such as VP16.

The F99S mutation is not localized in an enzymatic domain of the protein that would modify its activity, but rather in a region previously shown to be involved in the interaction of XPB with the p52 subunit of TFIIH, its regulatory partner in the complex^11^. The partnership between XPB and p52 results in an increase in the ATPase activity of XPB, but we excluded that a modification of this activity is involved in chromatin decondensation observed here because chromatin unfolding was observed with or without cellular ATP depletion. In addition, the fact that XPB^F99S^ induces the same chromatin decondensation as the NTD deletion mutant XPB^320–782^ indicates that the cause is more likely to be related to the integrity of the NTD and deregulation of the XPB/KAT2A partnership, rather than modification of XPB activity that induces chromatin decondensation.

Mutations and transcriptional deregulation of several global genome-organizing complexes are linked to global alterations in chromatin structure and have emerged as key players in human diseases^31^. Some HAT complexes not only carry out gene-specific regulatory functions, but also exhibit global chromatin modifying functions by regulating higher chromatin organization that have an impact in human diseases^24^. For instance, it has been shown that polyQ expansions in the hSAGA subunit ATXN7 deregulate KAT2A activity, impact chromatin condensation and result in spinocereballar ataxia^32^. Our results go further and show that a mutation in a gene coding for a subunit of a basal transcription factor may also have an impact on the HAT activity of KAT2A in *trans*, leading to global alterations in chromatin structure and a human genetic disorder. The precise mechanism by which TFIIH-XPB^F99S^ increases KAT2A HAT activity is yet to be fully understood, but according to the results presented in this study, it is reasonable to postulate that TFIIH-XPB^F99S^ might trigger higher-order chromatin decondensation by prolonging the binding and recruitment of KAT2A-containing HAT complexes to chromatin. F99S could also overcome negative regulation of KAT2A by XPB NTD that occurs in normal conditions. The absence of this regulation would induce unscheduled chromatin unfolding, similar to some BRCA1 mutants^29^.

The higher-order organization of the genome into chromatin fibers and chromosomes is well known to critically contribute to gene regulation^33^. There is an increasing list of diseases in which changes to histone modifications and transcription dysregulation have been documented^31^, among which XP-B/CS can now be included.

Upregulation of gene expression could be the result of several mechanisms, including increased stability of mRNA or sequestration in the nucleus due to the cellular stress generated by a mutation that alters the rate of mRNA transcription^34^. The upregulated genes in XP-B/CS^F99S^ cells are probably over-transcribed, because the newly synthetized pre-mRNAs are also increased. We compared our results with an earlier study in which the expression profiles of a limited number of cDNA sequences were determined in the clone 14 used in our study^35^. We observed a strong correlation between these data, as 80% of the genes previously identified as overexpressed were also identified in our study. We confirmed these data for a few representative genes, showing that the F99S mutation is directly involved in the inappropriate gene overexpression observed in the patient cells. In our study, the level of pre-mRNA for some of these genes, such as NETO1, was almost undetectable in the stably transfected cells, indicating that they are silenced in wild-type fibroblasts and strongly de-repressed in patient-derived fibroblasts.

Acetylated H3K9 is directly linked to the opened chromatin state that is transcriptionally permissive, while methylated H3K9 mediates gene silencing^36^. Accordingly, there is a global increase in acetylated H3K9 in patient-derived cells while methylated H3K9 is decreased. Along the same lines, the overexpressed gene promoters and to a lesser extent the neighboring sequences have sequestrated KAT2A and hyperacetylated H3K9 in XP-B/CS^F99S^ cells. Knocking down KAT2A activity resulted in a return to normal levels of expression of these promoters, together with hypoacetylation of H3K9 and decrease of TICs. Interestingly enough, knocking down KAT2A HAT activity affected H3K9ac close to the *LRRC42* promoter but hardly impaired its pre-mRNA synthesis or the recruitment of the TIC to its promoter. These data show that although the KAT2A HAT affects global chromatin structure, it only affects the expression of a specific group of genes in XP-B/CS^F99S^ patient-derived fibroblasts. This implies that global chromatin acetylation and higher-order chromatin architecture may not be the only determinants of gene regulation in cells. Thus, additional events, that need to be determined, are probably needed to increase gene expression in the decondensed chromatin of patient-derived cells.

The XPB^F99S^ mutation was found in two brothers who showed classical clinical signs of DNA repair defects, with severe sun sensitivity and freckling^20^. There is increasing evidence that higher-order chromatin structure plays a key role in the efficiency of DNA repair^37^. However, our previous results demonstrated that the NER defect in XP-B/CS^F99S^ cells is causally related to reduced XPB ATPase activity due to a lack of interaction between XPB^F99S^ and its regulatory TFIIH subunit p52^11^. In line with this view, we did not observe recruitment of KAT2A to a locally UV-irradiated area of the nucleus and did not observe an impact of KAT2A knockdown on the recruitment of the DNA lesion recognition factor XPC to damaged DNA in wild-type fibroblasts (Supplementary Fig. 7).

Besides DNA repair-related phenotypes, XP-B/CS^F99S^ patients display additional clinical signs, such as a diminished stature, hearing and movement impairment, and neurological degeneration^38^ that may be related to the deregulation of transcription. Gene ontology analysis showed that the upregulated genes in patient-derived fibroblasts belong to several fundamental cellular processes, such as development, metabolism, and response to a stimulus that may all be linked to these non-DNA repair phenotypes. Several studies have revealed that human diseases could be the result of inappropriate transcriptional activation/de-repression^39^. Interestingly, the phenotypes of these diseases are tissue-specific, as are the XP-B/CS phenotypes. It therefore seems likely that some of the XP-B/CS phenotypes are caused by the overexpression of a few specific genes that are normally expressed exclusively in particular tissues, or only under certain conditions that remain to be established. Finally, we analyzed the expression of several pre-mRNAs in XP-D/CS or CS-B patient cells mutated in XPD and CSB, respectively, and found that some genes, such as *RARβ2* or *RNF130*, were also overexpressed in these cells (Supplementary Fig. 8). These observations suggest that deregulation of the transcriptional program in XP-B/CS cells is not restricted to the F99S mutation and could be a general cellular phenotype of CS patients. In any case, the identification of the molecular defect associated with the inappropriate expression of genes in XP and CS patients may pave the way for new treatments that are based on drugs targeting transcription overexpression and that will relieve some of their symptoms.

## Methods

### Cell lines and reagents

The A0-3 cell line was cultured in DMEM/HAMF12 supplemented with 5% FCS and penicillin 100 UI/ml and streptomycin 100 μg/ml. The U2OS17 cell line was cultured in DMEM (4.5 g/l Glucose) supplemented with 10% fetal calf serum (FCS) and Gentamycin (40 μg/ml). The U2OS17 stable cell line is one of the clones generated by the team of Dr. Soutoglou^13,17,18^. Briefly, U2OS cells were transfected with a plasmid that contains an I-SceI recognition site flanked by 256 copies of the lac operator (lacO) on one side and by 96 copies of the tetracycline response element on the other side (tetO). There are two insertions of LacO repeats in the U2OS17 cells, and consequently the following number of spots that can be observed depends on the cell cycle stage: two spots in G1, four spots in G2, three spots in the middle of S phase. Note also that in some cases the two spots are very closed to each other.

Stable U2OS17 cells expressing lacR fusion proteins were produced as follows. U2OS17 cells were stably transfected with pEGFP-LacR-XPB^WT^ or pEGFP-LacR-XPB^F99S^ and selected with G418 500 µg/ml and Hygromycin B 200 µg/ml. Clones were selected and cultured in DMEM (4.5 g/l glucose) supplemented with 10% fetal calf serum (FCS), Gentamycin (40 mg/ml), IPTG 5 mM, G418 500 µg/ml, and Hygromycin B 200 µg/ml.

XP-B/CS^F99S^ cells (XPCS1BA-SV40)^20^ expressing *ERCC3* c.296T>C mutant, pPhe99Ser (RefSeq accession number NM_000122.1) and the stably transfected XP-B/CS^F99S^ + XPB^WT^ cells are SV40-transformed human fibroblasts that were cultured in DMEM/HAMF10 supplemented with 10% FCS.

Clones 5 and 14 are XPCS1BA-SV40 cells that were stably transfected with a vector that expresses ERCC3 c.355A>C, pThr119P mutant^27^. Clone 5 expresses only the XPB^T119P^ mutant and clone 14 only XPB^F99S^.

XP-D/CS^G602D^ cells (XPCS2)^40^ are human primary fibroblasts that express *ERCC2* c.1805G>A mutation (RefSeq NM_000400.3).

CS-B cells (CS1ANSV)^41^ are SV40-transformed human fibroblasts that express CSB c.1088A>T mutation (RefSeq NM_001277058.1).

All these cells were cultured in DMEM/HamF10 (1:1) medium containing 10% FCS and 40 mg/ml gentamycin at 37 °C in a 5% CO_2_ incubator.

A complete list of reagents, oligonucleotides, siRNA, plasmids, and chemicals used in this manuscript is available in Supplementary Table 1.

### CRISPR/Cas9 engineered U2OS^XPB::GFP^ cells

U2OS^XPB::GFP^ cells expressing XPB-GFP from the XPB locus were prepared by genome editing with the CRISPR-Cas9 system, as follows. A guide RNA sequence targeting cleavage next to the STOP codon of the XPB gene was cloned into the pMLM3636 expression plasmid (targeted sequence, with guide RNA spacer sequence in uppercase and PAM in lowercase 5′AGGAAATGATGCTTAGGCAggg3′). A donor plasmid was constructed with eGFP-2A-puromycin coding sequences inserted at the position of the XPB STOP codon and flanked by the 5′ homology arm chr2:127257599-127257872 (hg38) and 3′ homology arm chr2:127257596-127256813 (hg38). Guide RNA, Cas9 and donor plasmids were electroporated into U2OS cells using the Amaxa nucleofector II with kit V and program X-001 (Amaxa, Lonza, Switzerland). Clones of cells expressing XPB-GFP-2A-puromycin were selected with puromycin and targeted insertion was confirmed by PCR amplification of the expected junction fragments. A clone that expresses XPB-GFP but no longer expresses XPB (due to the modification of all XPB gene alleles) was used for further experiments. U2OS cells were purchased from ATCC and checked for the absence of mycoplasma contamination.

### Constructs

Full-length XPB coding sequence was amplified and ligated into the EcoRI/BamHI restriction sites of the pEGFP-N1 vector (Clonetch) giving the pEGFP-XPB construct. To generate fusions between LacR, XPB, and GFP, the cDNA of LacR was amplified by PCR and cloned in pEGFP-XPB vector between GFP and XPB. The FLAG-KAT2A-expressing construct was described in^42^.

### Immunofluorescence and 3D reconstructions

Staining was done following a standard IF protocol. Briefly, cells were washed with PBS, fixed in freshly prepared 4% paraformaldehyde (PFA) for 10 min at RT and permeabilized with PBS 0.1% Triton X-100 for 3 × 5 min at RT. Blocking (RT, 20 min) and incubations with antibodies (RT, 1 h) were performed with 10% heat-inactivated FCS in PBS 0.1% Triton X-100 and washes were done with PBS 0.1% Triton X-100 at RT for 3 × 5 min. Nuclei were counterstained with freshly prepared 1 μg/mL DAPI in PBS for 2 min at RT and cells were mounted using the ProLong Gold antifade reagent of Molecular Probes. Confocal microscopy pictures were taken with a Leica SP2 microscope; Z stack width was usually 0.5 μm. For counting colocalised signals, at least 100 cells were analyzed for each condition. For 3D reconstructions, stacks were captured using a Leica DM6000 microscope with a Leica CSU22 spinning disc and an Andor Ixon 897 camera, the step size was 0.2 μm. For reconstruction of the images and the quantification of the volume filled by the signal, Imaris Software (Bitplane) was used. Results are shown in box and whisker plots. *p*-values represent significant differences based on a Kruskal–Wallis test.

### Immunofluorescent-based DNA lesion quantification

Five thousand cells were plated in 96-well plates (OptiPlates-96, Perkin Elmer). 24 h later, cells were UV-irradiated with a UV-C lamp (30 J/m^2^) for the indicated period of time at 37 °C, 5% CO_2_ and recovered in fresh medium. Immuno-labeling of (6-4)PP was performed using the mouse 64M-2 antibody following the IF protocol. A step of DNA denaturation was added after permeabilization where cells were treated with freshly prepared 2M HCl for 30 min at RT. Cells were washed with PBS for 5 min and blocked. (6-4)PP lesions were quantified using an IN Cell Analyser 1000 imaging system (GE Healthcare) and the amount of (6-4)PP removal was determined.

### Cell extract and western blot

Cells were scraped in PBS on ice, lysed in RIPA buffer 10 mM Tris-Cl (pH 8.0), 1 mM EDTA, 0.5 mM EGTA, 1% Triton X-100, 0.1% sodium deoxycholate, 0.1% SDS, 140 mM NaCl for 30 min at 4 °C and centrifuged at 18 kg for 20 min at 4 °C. Fifteen micrograms of each extract was loaded on an SDS-PAGE gel.

### Purification of rKAT2A and the rHAT-ATAC module

Recombinant FLAG tagged KAT2A was purified using anti-FLAG M2-agarose beads (Sigma). M2-agarose-bound protein complexes were washed three times with immunoprecipitation (IP) buffer (25 mM Tris-HCl, pH 7.9, 10% (v/v) glycerol, 0.1% Nonidet P-40, 0.5 mM DTT, 5 mM MgCl_2_) containing 0.5 M KCl and twice with IP buffer containing 100 mM KCl. After washing, proteins were eluted with a 1000 × excess of the flag epitope peptide. The reconstituted human HAT module of the ATAC complex (rHAT-ATAC) containing the KAT2A, ADA2a, ADA3, and SGF29 subunits was expressed and purified from insect cells^25^.

### Histone acetyltransferase assay

The HAT activity of rKAT2A or rHAT-ATAC was measured using histone acetyltransferase assay^25^. Recombinant histones H3.3 were incubated with rKAT2A or the rHAT-ATAC module in the presence of acetyl-CoA in HAT buffer (50 mM Tris-HCl pH 8.0, 7% glycerol, 25 mM NaCl, 0.1 mM EDTA, 5 mM DTT) for 1 h at 30 °C. The reaction was then analyzed by western blotting with specific antibodies (H3 and H3K9ac).

### Pull-down assay

XPB was expressed in baculovirus and incubated for 4 h at 4 °C with anti-XPB covered beads in lysis buffer (20 mM Tris-HCl pH 7.5, 150 mM KCl, 20% glycerol, 0.1% NP40). Following three washes with lysis buffer, beads were further incubated at 4 °C for 1 h with 500 ng of purified Flag-KAT2A in lysis buffer. Pull downs were washed with RIPA buffer and analyzed by western blotting.

### Reverse transcription and real-time quantitative PCR

Total RNA was isolated using TRI REAGENT (MRC) and purified by phenol-chloroform extraction. RNA was reverse transcribed with SuperScript IV reverse transcriptase (Invitrogen) and Oligo d(T). Quantitative PCR was done using the SYBR Green Master Mix in a Lightcycler 480 (Roche). mRNA levels were normalized against the housekeeping *GAPDH* mRNA. Sequences of primers are available in Supplementary Table 1.

### RNA-seq analysis

Total RNAs from XP-B/CS and XP-B/CS + XPB^WT^ cells were extracted using GenElute Mammalian Total RNA Miniprep Kit (Sigma). Libraries were prepared with TruSeq Stranded mRNA Sample Preparation Kit following guide instructions and subsequently analyzed on an Illumina Hiseq 4000 as single-end 50 base reads following Illumina’s instructions. Image analysis and base calling were performed using RTA 2.7.3 and bcl2fastq 2.17.1.14. Reads were mapped onto the hg19 assembly of the human genome. Read counting was performed with HOMER v4.8.3(65) and expression was estimated with EdgeR. Genome ontology was performed with The Database for Annotation and Integrated Discovery (DAVID) v6.7 (https://david.ncifcrf.gov).

### Chromatin immunoprecipitation (ChIP)

Cells were cross-linked at room-temperature (RT) for 10 min with 1% formaldehyde. Chromatin was prepared and sonicated on ice for 30 min using a sonicator (Q800R; Qsonica)^28^. Protein G Sepharose beads (Upstate) were prepared by incubation with antibodies at 4 °C for 2 h and incubated with samples at 4 °C overnight. After washing, protein-DNA complexes were eluted and decrosslinked. DNA fragments were purified using Qiaquick PCR purification Kits (QIAGEN) and analyzed by qPCR using the set of primers indicated in Supplementary Table 1.

### siRNA

The ON-TARGET plus smart pool siRNA control or human KAT2A targeting siRNAs were purchased from Dharmacon and transfected at a final concentration of 100 nM using the Lipofectamine RNAiMax reagent (Invitrogen) following the manufacturer’s protocol.

### Quantification and statistical analysis

Statistical analysis of experimental data was performed using a Student’s paired *t* test for ChIP and RT-PCR, and a Kruskal–Wallis test for 3D reconstruction analysis. Results are presented as mean ± standard deviation (SD). *p*-values are shown.

### Reporting Summary

Further information on experimental design is available in the Nature Research Reporting Summary linked to this article.

## Supplementary information

Supplementary Information

Description of Additional Supplementary Files

Supplementary Data 1

Reporting Summary

Source Data

## Data Availability

The data that support the findings of this study are available from the corresponding author upon request. The RNA-seq data reported in this paper is accessible at the GEO under accession code GSE125963.
