# Supplementary material for: Functional interplay between TFIIH and KAT2A regulates higher-order chromatin structure and class II gene expression
Source: Nat Commun. 2019 Mar 20;10:1288. doi: 10.1038/s41467-019-09270-2 (PMC6426930; doi:10.1038/s41467-019-09270-2)
Supplement: Supplementary file 1 — Supplementary Information [file 41467_2019_9270_MOESM1_ESM.pdf]

Functional interplay between TFIIH and KAT2A regulates higher-order chromatin structure and class II gene expression

Jérémy Sandoz [1,2,3,4], Zita Nagy [1,2,3,4], Philippe Catez [1,2,3,4], Gizem Caliskan [1,2,3,4] , Sylvain Geny [5], Jean-Baptiste Renaud [5], Jean-Paul Concordet [5], Arnaud Poterszman [1,2,3,4], Laszlo Tora [1,2,3,4], Jean-Marc Egly [1,2,3,4], Nicolas Le May [1,2,3,4] and Frédéric Coin [1,2,3,4,6]

[1] Institut de Génétique et de Biologie Moléculaire et Cellulaire Illkirch Cedex, C.U. Strasbourg, France

[2] Centre National de la Recherche Scientifique, UMR7104, 67404 Illkirch, France

[3] Institut National de la Santé et de la Recherche Médicale, U1258, 67404 Illkirch, France

[4] Université de Strasbourg, 67404 Illkirch, France

[5] Laboratoire Structure et Instabilité des Génomes, INSERM U1154, CNRS UMR7196, Muséum national d'Histoire naturelle, 43 rue Cuvier, 75005 Paris, France

[6] Corresponding author, fredr@igbmc.fr

Tel: +33 3 88 65 34 49

Supplementary Data

Supplementary Data includes 8 Supplementary Data files (Figure 1 to 8) and 1 Supplementary Table (Table 1):

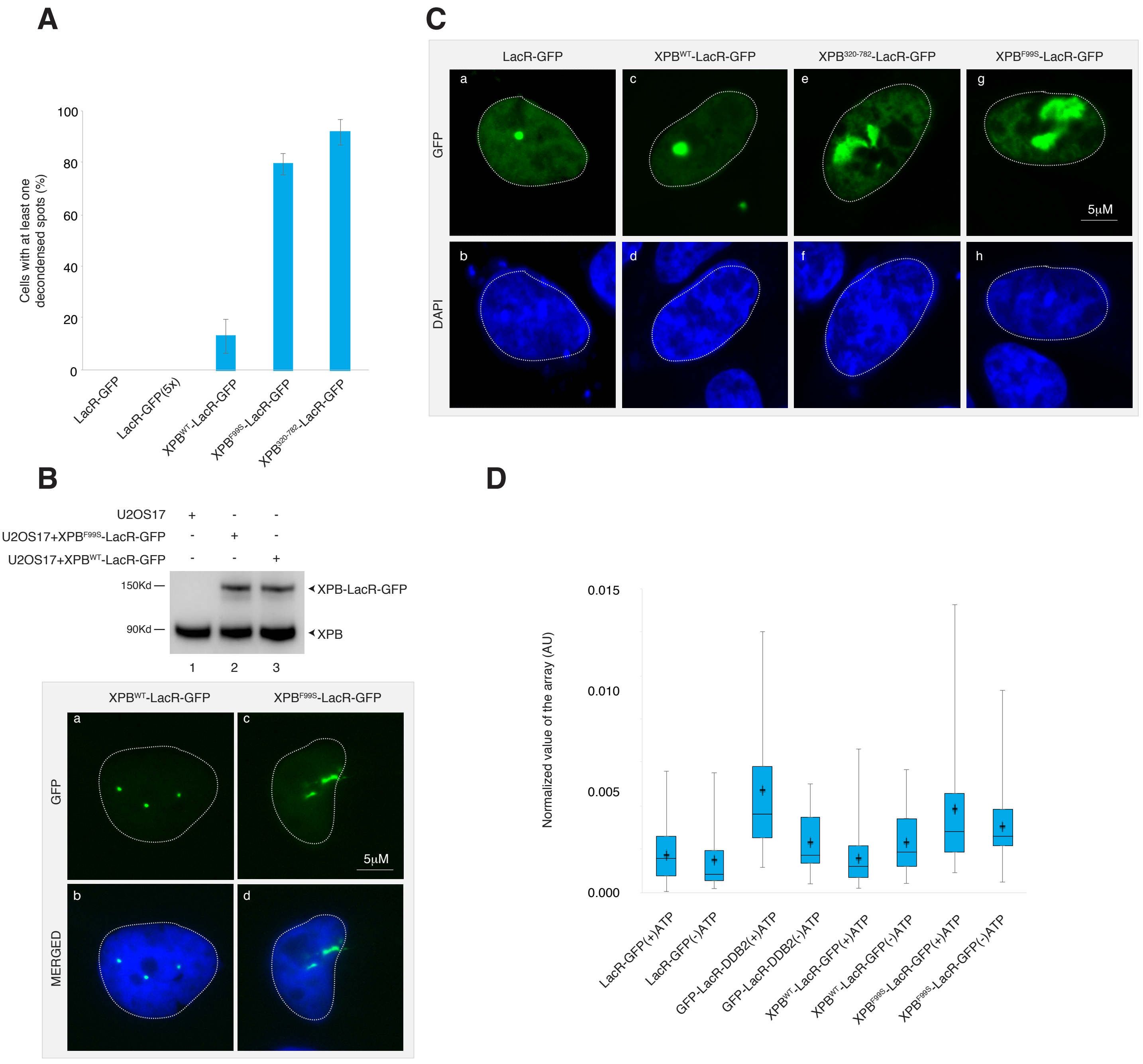

Supplementary Figure 1. **(A).** Quantification of the percentage of cells with at least one decondensed array after tethering of the indicated fusion proteins. Values represent the mean of two independent experiments (*n*=50 cells). **(B)**. **Upper panel**; Stable U2OS17 cells expressing either XPB^{WT}-LacR-GFP or XPB^{F99S}-LacR-GFP were established and total cell extracts were resolved by SDS-PAGE and immunoblotted with mouse-monoclonal anti-XPB. **Lower panel**; In stable U2OS17 cells expressing XPB^{WT}-LacR-GFP (a-b) or XPB^{F99S}-LacR-GFP (c-d), GFP was detected by fluorescence microscopy and cells were stained with DAPI. **(C)**. LacR-GFP (a-b), XPB^{WT}-LacR-GFP (c-d), XPB$^{320-782}$-LacR-GFP (e-f) and XPB^{F99S}-LacR-GFP (g-h) were transfected in hamster A0-3 cells and tethered to chromatin. GFP was detected by fluorescence microscopy 24 hrs post transfection. **(D)**. LacR-GFP, GFP-LacR-DDB2, XPB^{WT}-LacR-GFP or XPB^{F99S}-LacR-GFP were transfected in U2OS17 cells incubated in medium with or without inhibitor of ATP synthesis. The Whisker box plot represents the quantifications of the relative array volumes (volume of the array/volume of the nucleus; 500 cells for each condition; normalized value of the array).

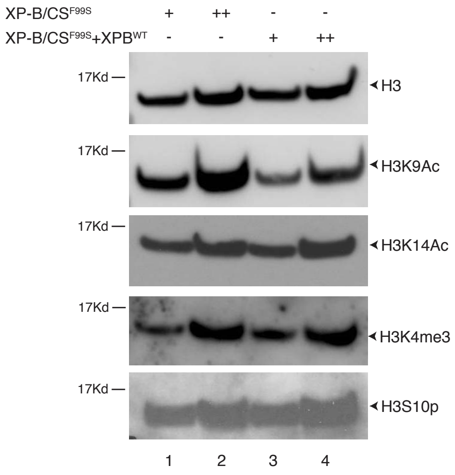

Supplementary Figure 2. Histones were extracted from either XP-B/CS^{F99S} or XP-B/CS^{F99S}+XPB^{WT}, resolved by SDS-PAGE and immunoblotted with either polyclonal rabbit anti-histone H3, monoclonal mouse anti-histone H3K9Ac, polyclonal rabbit anti-histone H3K14Ac, monoclonal mouse anti-histone H3K4me3 or polyclonal rabbit anti-histone H3S10p.

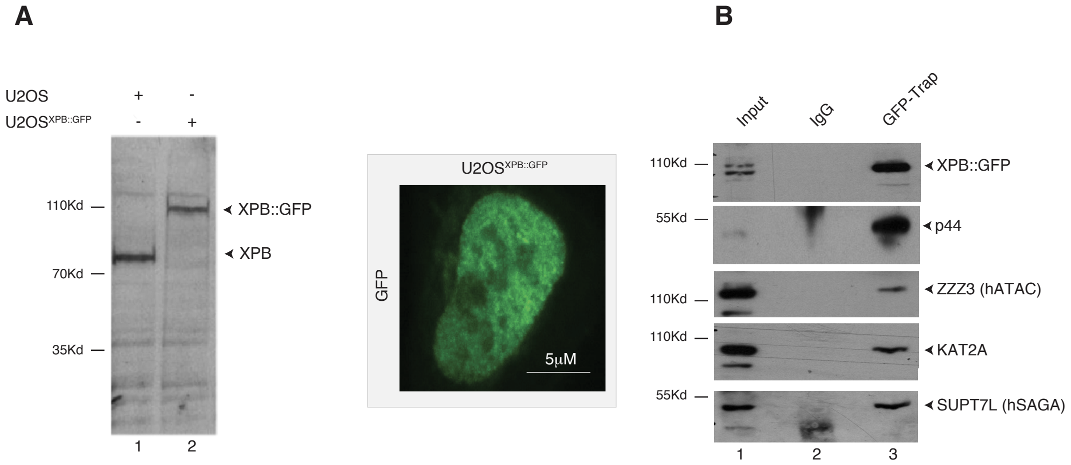

Supplementary Figure 3. (A). Nuclear extracts from U2OS or U2OS$^{XPB::GFP}$ were resolved by SDS-PAGE and immunoblotted using polyclonal rabbit anti-GFP. Alternatively, U2OS$^{XPB::GFP}$ cells were fixed and GFP detected by fluorescent microscopy **(B).** TFIIH was immunoprecipitated from nuclear extracts of U2OS$^{XPB::GFP}$ cells using anti-GFP antibody (GFP-Trap ®) and washed with 200mM salt. The control IP was performed with unspecific anti-IgG. Proteins on the resin were resolved by SDS-PAGE and immunoblotted using polyclonal rabbit anti-GFP, monoclonal mouse anti-p44 (TFIIH), polyclonal rabbit anti-KAT2A, polyclonal rabbit anti-ZZZ3 (hATAC) or polyclonal rabbit anti-SUPT7L (hSAGA) antibodies.

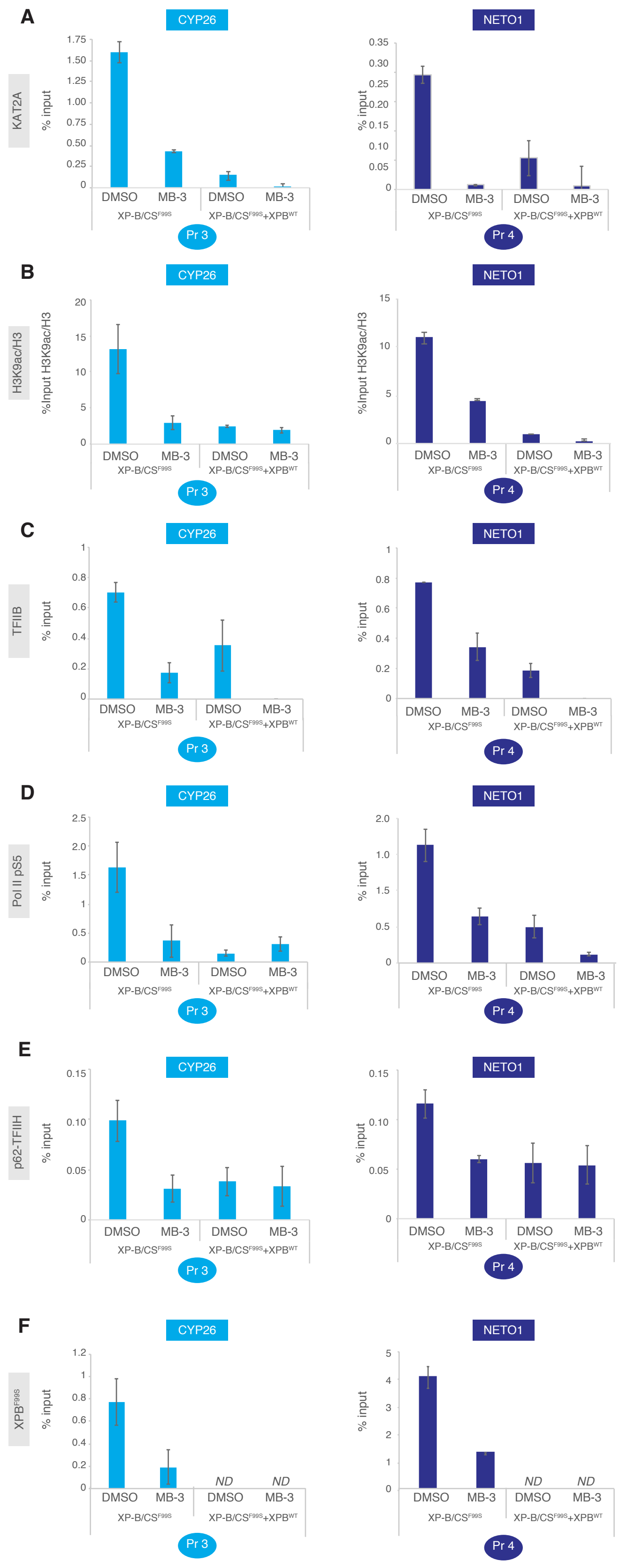

Supplementary Figure 4. **(A-D)**, ChIP monitoring the occupancy of: KAT2A (A), H3K9ac/H3 (B), TFIIB (C) and Pol II pS5 (D) on the promoter (Pr) regions of *CYP26* (Pr 3) and *NETO1* (Pr 4) genes in XP-B/CS^{F99S} and XP-B/CS^{F99S}+XPB^{WT} treated either with DMSO or MB-3. **(E-F),** ChIP monitoring the occupancy of p62 (E) or XPB^{F99S} (F) TFIIH subunits on Pr 3 and 4 regions of *CYP26* and *NETO1* genes in XP-B/CS^{F99S} treated either with DMSO or MB-3.

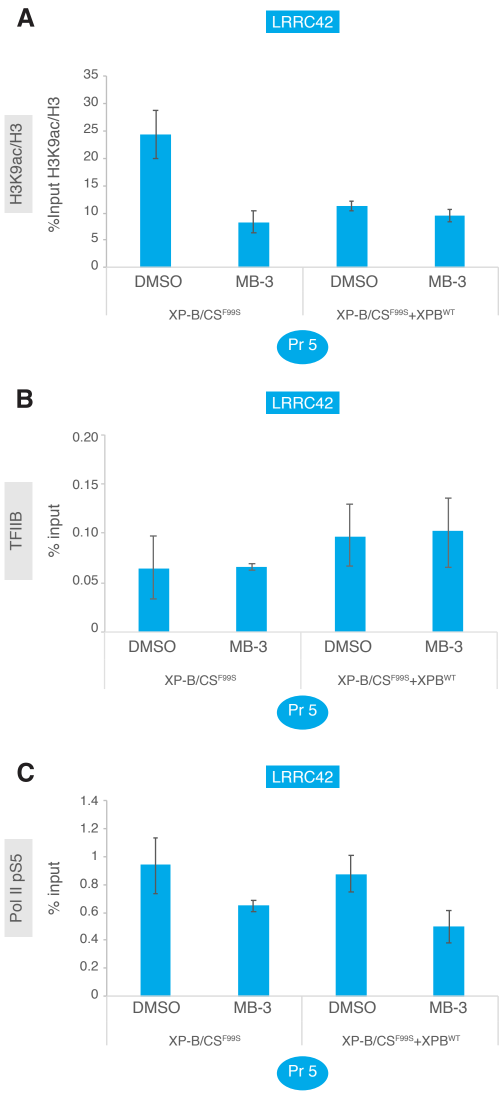

Supplementary Figure 5. **(A-C)**, ChIP monitoring the occupancy of: H3K9ac/H3 (A), TFIIB (B) and Pol II pS5 (C) on the promoter (Pr5) region of the *LRRC42* gene in XP-B/CS^{F99S} and XP-B/CS^{F99S}+XPB^{WT} treated either with DMSO or MB-3.

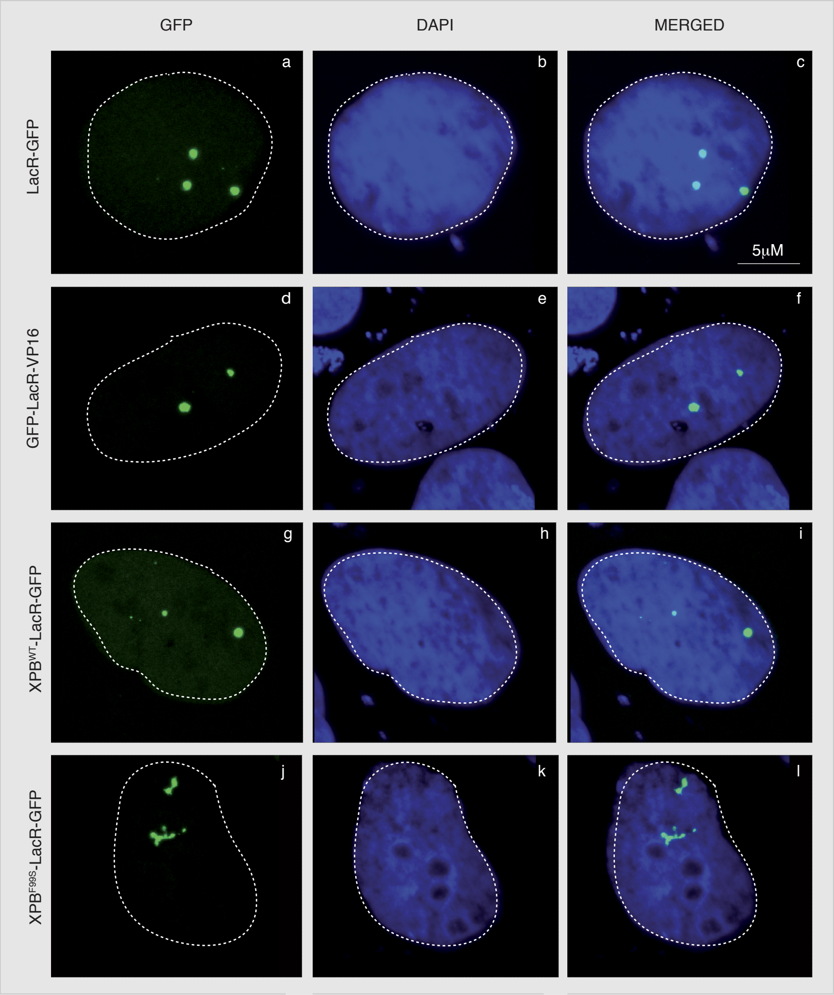

Supplementary Figure 6. LacR-GFP (a-b-c), GFP-LacR-VP16 (d-e-f) [12], XPB^{WT}-LacR-GFP (g-h-i) and XPB^{F99S}-LacR-GFP (j-k-l) were transfected in human U2OS17 cells and tethered to chromatin. GFP was detected by fluorescence microscopy 24 hrs post transfection and cells were stained with DAPI.

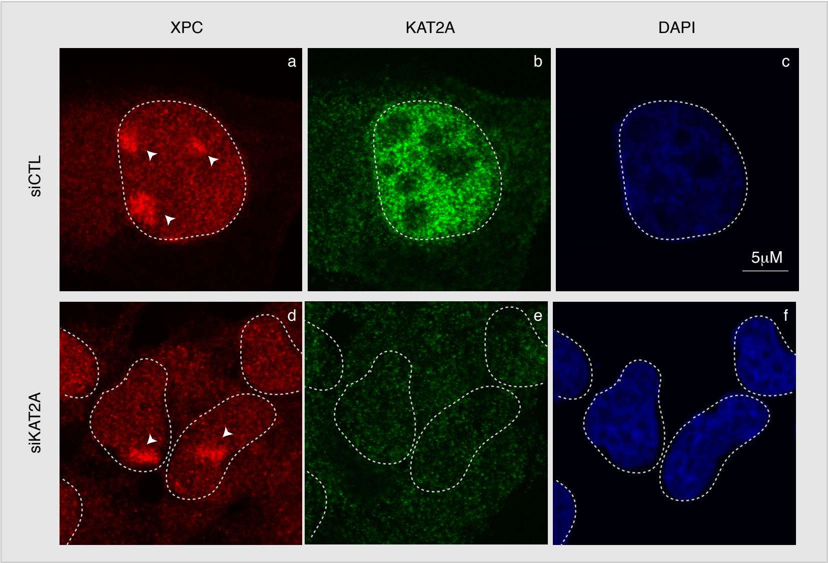

Supplementary Figure 7. Following treatment with siCTL or siKAT2A as performed in Figure 5, wild-type fibroblasts were locally UV-irradiated (100 J/m^2), fixed after 15 minutes and co-stained with rabbit polyclonal anti-XPC (a-d) and polyclonal rabbit anti-KAT2A (b-e) (for reference on local UV irradiation see [23]). Arrows indicate locally irradiated areas. Treatment with siKAT2A depletes KAT2A from cell nucleus but does not impair the recruitment of XPC to local irradiated areas.

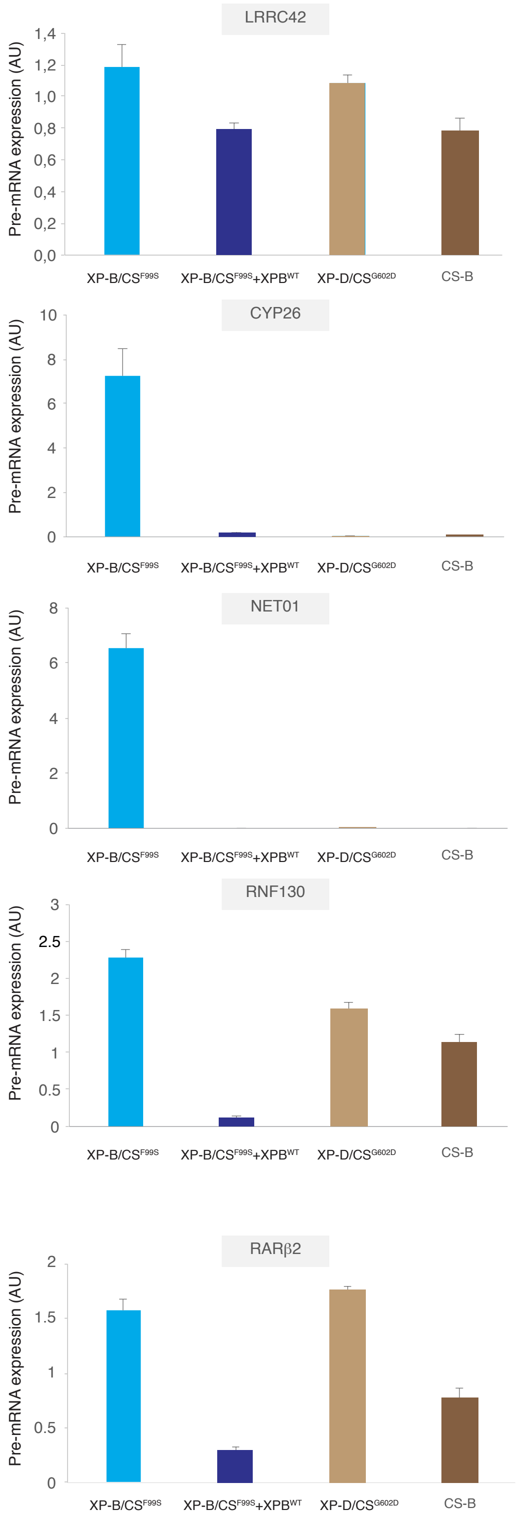

Supplementary Figure 8. Pre-mRNA level (+/- SD, three independent experiments) of *LRRC42, CYP26*, *NETO1*, *RNF130 and RARβ2* was analyzed in XP-B/CS^{F99S}, XP-B/CS^{F99S}+XPB^{WT}, XP-D/CS^{G602D} and CS-B cells. Data represent the relative expression levels of the pre-mRNA relative to GAPDH mRNA.

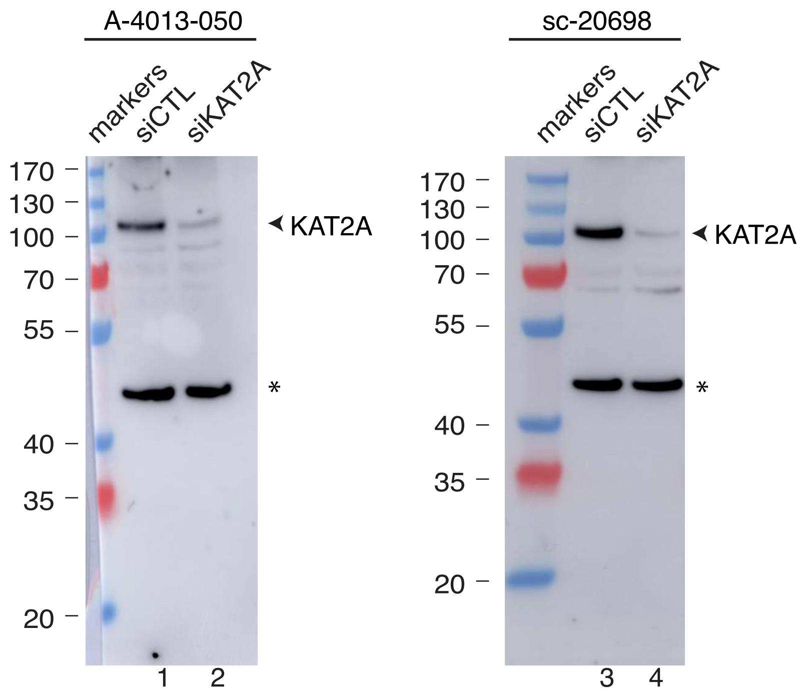

Supplementary Figure 9: MRC5 fibroblasts were transfected with siCTL or siKAT2A for 24hrs and 50 μg of cell extracts were resolved by SDS-PAGE and immuno-blotted with the indicated Ab against KAT2A.

Supplementary Table 1: Reagent and resource used in the associated manuscript

REAGENT or RESOURCE	SOURCE	IDENTIFIER
**Antibodies**		
Monoclonal mouse anti-(6-4) photoproducts (64M-2)	Cosmo Bio	CAC-NM-DND-002
Polyclonal rabbit anti-H3	Abcam	ab1791
Monoclonal mouse anti-H3K9Ac	Active Motif	61251
Polyclonal rabbit anti-H3K9me2	Cell signaling	9753S
Polyclonal rabbit anti-H3K14Ac	Active motif	39599
Monoclonal mouse anti-H3K4me3	Abcam	ab1012
Polyclonal rabbit anti-H3S10P	Diagenode	CS-116-100
Polyclonal rabbit anti-H3Ac	Upstate	06-599
Polyclonal rabbit anti-GFP	Torrey Pines Biolabs	TP401
Polyclonal rabbit anti-XPB	Santa Cruz	sc-293
Monoclonal mouse anti-XPB	IGBMC Antibody Facility	1B3
Monoclonal mouse anti-p62	IGBMC Antibody Facility	3C9
Polyclonal rabbit anti-p62	Santa Cruz	sc-292
Monoclonal mouse anti-p52	IGBMC Antibody Facility	5D6
Monoclonal mouse anti-p44	IGBMC Antibody Facility	1H5
Monoclonal mouse anti-KAT2A	IGBMC Antibody Facility	2A6
Polyclonal rabbit anti-KAT2A	Santa Cruz	sc-20698, see Supplementary Figure 8
Polyclonal rabbit anti-KAT2A (ChiP and IF)	Epigentek	A-4013-050, see Supplementary Figure 8
Polyclonal rabbit anti-XPC	Bethyl	A301-122A
Polyclonal rabbit anti-Beta Tubulin	Abcam	ab6046
Monoclonal mouse anti-Actin	IGBMC Antibody Facility	2D7
Polyclonal rabbit anti-CDK7	Invitrogen	PA1-29162
Polyclonal rabbit anti-TFIIB	Santa Cruz	sc-225
Polyclonal rabbit anti-SUPT7L	Bethyl Lab	A302-803A
Polyclonal rabbit anti-WDR5	Merck Millipore	07_706
Polyclonal rabbit anti-ZZZ3	IGBMC Antibody Facility	2616
Polyclonal rabbit anti-KAT2B	Abcam	ab12188
Polyclonal rabbit anti-Flag	Sigma	F7425
Monoclonal rat anti-Pol II Sp5	Active Motif	61085
Normal rabbit IgG	Santa Cruz	sc-2345
Peroxidase-conjugated AffiniPure Donkey Anti-Mouse IgG	Jackson Immuno Research	715-035-151

Peroxidase-conjugated AffiniPure Goat Anti-Rabbit IgG	Jackson Immuno Research	111-035-144
Alexa Fluor 546 goat anti rabbit IgG	Life technologies	A110035
Alexa Fluor 488 goat anti mouse IgG	Life technologies	A11001
**Chemicals, Peptides and Recombinant Proteins**		
G418 disulfate salt solution	Sigma Aldrich	G8168
Hygromycin B solution from Streptomyces hygroscopicus	Sigma Aldrich	H0654
IPTG	Euromedex	EU0008-A
Paraformaldehyde 16%	Electron Microscopy Sciences	15710
Butyrolactone (MB-3)	Sigma Aldrich	M2449
Spironolactone	Santa Cruz	sc-204294
KAT2A siRNA	Dharmacon	L-009722-02
Non targeting siRNA	Dharmacon	HUMAN-XX
DRB	Merck	287891
DAPI	Sigma	D9542
Triton X-100	Sigma	T8787
Tween 20	Sigma	P2287
SDS 20%	Euromedex	EU0660
Acrylamide 40%	Euromedex	EU0077-B
Temed	Serva	35925.01
APS	Thermo Fisher	17874
Sodium Azide	Aldrich	438456
2-Deoxy-D-glucose	Sigma	D8375
D-Glucose	Euromedex	UG3050
**Critical Commercial Assays**		
ProLong Gold Antifade Mountant	Invitrogen	P36930
PageRuler Plus Prestained Protein Ladder	Thermo Fisher	26619
Bis Tris	Sigma	B9754
NuPAGE MOPS SDS Running Buffer 20X	Novex	NP0001
NuPAGE LDS Sample Buffer 4X	Invitrogen	NP0007
SuperSignal West Pico PLUS Luminol/Enhancer Solution	Thermo Fisher	1863096
SuperScript IV reverse transcriptase	Invitrogen	18090050
TriReagent	Molecular Research Center	TR 118
GenElute Mammalian Total RNA Miniprep Kit	Sigma	RTN70
QIAquick PCR Purification Kit	Qiagen	28106
EpiQuick Total Histone Extraction Kit	Epigentek	OP-0006-100
GFP-trap_A	Chromotek	gta-20
G-coated Dynabeads	Thermo Fisher	10004D
Lipofectamine RNAiMAX	Thermo Fisher	13778
X-tremeGENE9	Roche	06365809001
LightCycler 480 SYBR Gren I Master	Roche	04887352001
Quantiect SYBR Green PCR Mix	Qiagen	204143
Protease Inhibitor Cocktail	Roche	11873580001

**Deposited Data**		
**Experimental Models: Cell Lines**		
U2OS17	Ziani et al.2014	Dr E. Soutouglou, IGBMC, France
U2OS17+XPB^{WT}-LacR-GFP	This paper	
U2OS17+XPB^{F99S}-LacR-GFP	This paper	
A03	Tumbar et al., 1999	
XP-B/CS^{F99S} (XPCS1BA)	Vermeulen et al., 1994	Dr. Wim Vermeulen, Erasmus University, Holland
XP-B/CS^{F99S}+XPB^{WT}	This study	
XP-B/CS^{F99S}+GFP	This study	
XP-D/CS^{G602D} (XPCS2)	Takayama et al., 1996	
CS-B (CS1ANSV)	Mayne et al., 1986	
Clone 14	Riou et al., 1999	Dr. Alain Sarasin, IGR, Paris, France
Clone 5	Riou et al., 1999	Dr. Alain Sarasin, IGR, Paris, France
**Oligonucleotides**		
**preRNA**		
LRRC42	GAAGTTTGGTGCT TTGCTCCC	ACTAAGCCCCAG AGCTTGACA
RARB2	GGCACACTGCTCA ATCTAGGT	TTGTCTTGCTAC CAATGCAGTTT
NETO1	TGGAATTAGTGGA GCCCGGA	CGGGGAGGGTA CTCACTGT
RNF130	GTGGTATTCAGAA AGCAGTTGACA	TTTGTGGATCAC AGCCCAGAT
CYP26	CCATGTGTCTGGC AGGACT	ATCACCGGCAC GTAGCAT
**RNA**		
GAPDH	GAGTCCACTGGC GTCTTCA	GGTCATGAGTCC TTCCACGA
LRRC42	CTTTGCTCCCTGT GTTTGCG	AAAGATCCAGGC AGGTCAGC
RARB2	ACCCCAGAACAAG ACACCAT	GCACTGAGAAG GCCTGTTTC

NETO1	CCGTCTTGGGAGT GCAAATT	ACTCACACGCTG GTAATGGT
RNF130	TGATCCACAAACC CGGTTCT	AAGCGGCCCGT GATATTTTC
CYP26	CGAGCACTCGTG GGAGAG	CCAAAGAGGAGT TCGGTTGA
**ChiP**		
LRRC42	CCTACCCCGAGCT TTTCG	AAGTAATGGGAG CCGGAGAG
RARB2	TGGTGATGTCAGA CTAGTTGGGTC	GCTCACTTCCTA CTACTTCTGTCA C
NETO1	GGGTGTGATGCG AGCGT	ATCCGGATGAGT CCGTCCT
RNF130	TGCCGGAGGGAC GATGA	CCACAGGCTGC AGGTCA
CYP26	TAAAGATTTTGGG CAGCGCC	CATCTGCAAGGT TTCCCCAA
Upstream RAR	AGGGACACGGTC TCACAGTACAAA	GCTTTGGTTCCT TCTGTTCTTCTC AG
**Recombinant DNA**		
pEGFP-LacR	Ziani et al., 2014	
pEGFP-LacR-XPB^{WT}	Ziani et al., 2014	
pEGFP-LacR-XPB^{F99S}	This paper	
pEGFP-LacR-XPB^{T119P}	This paper	
pEGFP-LacR-XPB$^{320-782}$	This paper	
pEGFP-LacR-XPB$^{1-550}$	This paper	
pCDNA3-FLAG-KAT2A		Dr L.Tora, IGBMC, France
pEGFP-lacR-DDB2		
**Software and Algorithms**		
Image J	NIH	N/A
Imaris Software	Bitplane	
The Database for Annotation and Integrated Discovery (DAVID) v6.7		https://david.ncifcrf.gov
HOMER v4.8.3(65)		http://homer.ucsd.edu/homer
**Other**		
Sonicator Q800R	Qsonica	
Ligthcycler 480	Roche	
In Cell Analyzer 1000 imaging system	GE Healthcare	N/A
Leica DM6000 microscope	Leica	DM6000
Leica CSU22 spinning disc	Leica	
Andor Ixon 897 camera	Andor Ixon	

OptiPlates-96	Greiner bio-one	655090
Lab-Tek Chambered Coverglass (4 wells)	Thermo Fisher	155383
UV-C lamp		
