# Supplementary material for: Functional interplay between TFIIH and KAT2A regulates higher-order chromatin structure and class II gene expression
Source: Nat Commun. 2019 Mar 20;10:1288. doi: 10.1038/s41467-019-09270-2 (PMC6426930; doi:10.1038/s41467-019-09270-2)
Supplement: Supplementary file 2 — Description of Additional Supplementary Files [file 41467_2019_9270_MOESM2_ESM.pdf]

Description of Additional Supplementary Files

File Name: Supplementary Data 1
Description: Relates to Figure 6b and is a comparative analysis of RNA-seq data from XP-B/CS^{F99S} and XP-B/CS^{F99S}+XPB^{WT} cells performed in triplicate.
